# Machine learning determines the incidence of Alzheimer’s disease based on population gut microbiome profile

**DOI:** 10.1093/braincomms/fcaf059

**Published:** 2025-04-15

**Authors:** Amedra Basgaran, Eva Lymberopoulos, Ella Burchill, Maryam Reis-Dehabadi, Nikhil Sharma

**Affiliations:** Department of Clinical and Movement Neurosciences, Queen Square Institute of Neurology, University College London, London WC1N 3BG, UK; Centre for Doctoral Training in AI-enabled Healthcare Systems, Institute of Health Informatics, University College London, London NW1 2DA, UK; King's College London, School of Medical Education, London WC2R 2LS, UK; Department of Clinical and Movement Neurosciences, Queen Square Institute of Neurology, University College London, London WC1N 3BG, UK; Department of Clinical and Movement Neurosciences, Queen Square Institute of Neurology, University College London, London WC1N 3BG, UK

**Keywords:** gut microbiome, microbiome, Alzheimer’s disease, dementia, machine learning

## Abstract

The human microbiome is a complex and dynamic community of microbes, thought to have symbiotic benefit to its host. Influences of the gut microbiome on brain microglia have been identified as a potential mechanism contributing to neurodegenerative diseases, such as Alzheimer’s disease, motor neurone disease and Parkinson’s disease (Boddy SL, Giovannelli I, Sassani M, *et al.* The gut microbiome: A key player in the complexity of amyotrophic lateral sclerosis (ALS). *BMC Med.* 2021;19(1):13). We hypothesize that population level differences in the gut microbiome will predict the incidence of Alzheimer’s disease using machine learning methods. Cross-sectional analyses were performed in R, using two large, open-access microbiome datasets (*n* = 959 and *n* = 2012). Countries in these datasets were grouped based on Alzheimer’s disease incidence and the gut microbiome profiles compared. In countries with a high incidence of Alzheimer’s disease, there is a significantly lower diversity of the gut microbiome (*P* < 0.05). A permutational analysis of variance test (*P* < 0.05) revealed significant differences in the microbiome profile between countries with high versus low incidence of Alzheimer’s disease with several contributing taxa identified: at a species level *Escherichia coli,* and at a genus level *Haemophilus and Akkermansia* were found to be reproducibly protective in both datasets. Additionally, using machine learning, we were able to predict the incidence of Alzheimer’s disease within a country based on the microbiome profile (mean area under the curve 0.889 and 0.927). We conclude that differences in the microbiome can predict the varying incidence of Alzheimer’s disease between countries. Our results support a key role of the gut microbiome in neurodegeneration at a population level.

## Teaser

Basgaran *et al*. reported that in countries with a high incidence of Alzheimer’s disease, the composition of the gut microbiome significantly differs, with lower diversity (*P* < 0.05). Using machine learning, they successfully predicted the population incidence of Alzheimer’s disease based on gut microbiome profile.

## Graphical Abstract

**Graphical Abstract:**
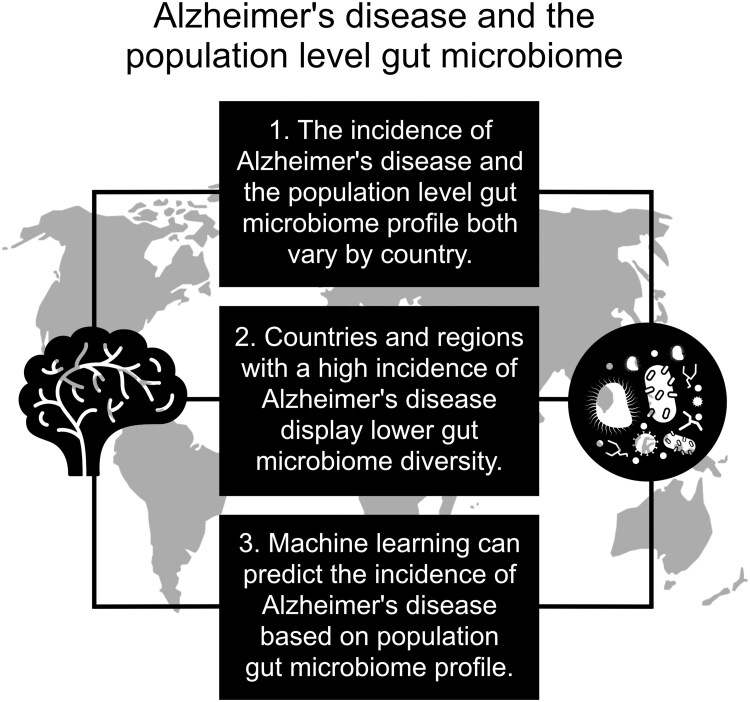

## Introduction

The human microbiome is a complex and dynamic community of microbes, thought to have symbiotic benefit to its host.^1,2^ It contains 10^14^ microbes from at least 1000 distinct microbial species.^3^ In comparison with the human genome that contains ∼20 000 human genes, there are tens of millions of microbial genes across thousands of species.^4^ Furthermore, the number of cells within the gut microbiome outnumbers human host cells by ∼10:1.^4^ Thus, given the scale and complexity of the microbiome, it is not surprising that the current literature increasingly suggests a substantial contribution to disease.

### The microglial axis

The link between the microbiome and the brain is hypothesized to involve microglia, otherwise known as the resident immune cells of the central nervous system (CNS).^5^ In a similar manner to peripheral macrophages, microglia play an important role in CNS homeostasis during development, adulthood and ageing.^6^ However, dysregulated microglia can cause neuronal damage and death directly, through the release of glutamate and cathepsins, or indirectly, through the release of TNF or reducing production of neuroprotective brain-derived neurotrophic factor (BDNF) and insulin-like growth factor.^7^ Increasing gut microbiota complexity promotes the maintenance of microglia under steady-state conditions. An absence of such complexity led to defects in microglial maturation, differentiation and function.^8^ Thus, by affecting microglia and reducing their immune capacity, a potential mechanism exists to explain how differences in the microbiome can contribute to neurodegeneration.

### Alzheimer’s disease

Alzheimer’s disease is a disorder of progressive neurodegeneration, with two defining histological features: neurofibrillary tangles and extracellular beta-amyloid peptide deposits within senile plaques in the CNS.^9^ Extensive work has elucidated an association between the composition of the gut microbiome and neurodegenerative Parkinson’s disease, precipitated by questions surrounding the significance of gastrointestinal symptoms in early-stage disease.^10,11^ However, Alzheimer’s disease has been less well explored. Recently, an increasing understanding of neuroinflammation as a key feature of Alzheimer’s disease has led to increasing research into the relationship between the gut microbiome and Alzheimer’s disease through the immune axis.^12^

From the microglial perspective, the link between microglia and Alzheimer’s disease is viewed as a double-edged sword. Initially, microglia sense and clear amyloid-beta; however, later microglial dysregulation leads to an inability to clear amyloid, and further amyloid deposition.^13^

Associations between the gut microbiome and Alzheimer’s disease have thus become an area of popular interest. Recent work has demonstrated that the gut microbiome of Alzheimer’s disease patients has decreased microbial diversity and is compositionally distinct from age and sex matched controls.^14^ Taxa differences are conflicting in previous studies, however. We aim to assess the reproducibility of these taxa differences in larger datasets with sample sizes of over 4000 patients combined. Furthermore, we investigate differences in microbiome diversity and assess whether machine learning can predict the incidence of Alzheimer’s disease based on population differences in the human gut microbiome.

## Methods

### Gut microbiome data

Two large, open-access microbiome data sets were used for our analysis, referred to as atlas1006 (Atlas) (*n* = 959)^15^ and curatedMetagenomicData (Curated) (*n* = 2012).^16^ 16S rRNA gene amplicon sequencing was the method used to study the gut microbiome in both datasets, and is most commonly used due to its high conservation, common expression on all bacterial sequences, low cost and simplicity to sequence.^17^ The threshold for defining membership in a given species was set at over 99% sequence similarity.

Sample analysis was restricted to those from healthy adult volunteers above the age of 18. Demographic data was explored, including gender, age and BMI, with the knowledge that such factors may influence gut microbiome composition and diversity.

### Burden of Alzheimer’s disease data

Data on the burden of Alzheimer’s disease were obtained from the Global Burden of Disease database for the years 2012 to 2017 inclusive.^18^ Metrics included rates of prevalence, incidence and disability-adjusted life years (DALYs) (per 100 000 individuals). We restricted analysis to industrialized countries with similar healthcare systems, to avoid confounding by differences in income of country.^19^

Geographical classification slightly differed between the two groups: the Atlas dataset grouped according to region such as Scandinavia, Central Europe, Southern Europe, UKIE and US; whilst the Curated dataset countries were selected to match the Atlas dataset and grouped according to country. The countries included are listed here by region: Scandinavia (Norway, Finland, Sweden), Central Europe (Netherlands, Belgium, Denmark, Germany), South Europe (Serbia, Italy, Spain, France), United States (US), UKIE (United Kingdom, Ireland), Luxembourg and Austria.

Regions and countries in these datasets were grouped based on Alzheimer’s disease incidence, into high and low disease groups and the microbiome profiles compared. Incidence was chosen as a marker of burden of Alzheimer’s disease as this is a better reflection of pathological onset and thus more helpful in testing a causative relationship with the microbiome. A threshold incidence of 92.5 cases per 100 000 individuals per year was used as a threshold to split the regions and countries into two similarly sized groups.

### Data analysis

All data processing and analysis was conducted in R [R version 4.0.2 (22 June 2020)] and used the *microbiome* (1.12.0), *phyloseq* (1.34.0), *SIAMCAT* (1.10.0) and *curatedMetagenomicData* (1.20.0) packages.^15,16,20-22^ The R markdown script is available on GitHub (via thesharmalab.com). Microbiome analyses were restricted to bacteria and did not include viruses and fungi. All analyses were performed on relative rather than absolute abundance of bacterial species. Analyses included alpha diversity, beta diversity, compositional abundance at a phyla level, PERMANOVA analysis at a species level and the SIAMCAT machine learning tool to determine the relationship between microbiome composition and Alzheimer’s disease burden. All analyses were compared by country, region and Alzheimer’s disease incidence group.

### Disease burden analysis

Rates of incidence, prevalence and DALYs (per 100 000 individuals) were plotted over time from the years 2012 to 2017, to confirm that there had been stability of all metrics and ensure allocation to groups did not change depending on the year chosen.

Heat maps of all three metrics for the year 2017 were then plotted to visualize differences in incidence, prevalence and DALY rates between the countries and regions.

Finally, a tree map of the incidence metric was plotted, using a threshold incidence of 92.5 cases per 100 000 individuals per year to split countries and regions into equally sized groups of high and low Alzheimer’s disease incidence.

#### Alpha diversity

Alpha diversity is a measure of diversity within the microbiome population and considers species richness (number of different species represented within a sample) or evenness (how evenly the different species are distributed in the sample) or both.^23^ Alpha diversity was quantified using the Shannon index, given its sensitivity to both species richness and evenness. The Wilcoxon signed rank test was used to assess for differences in alpha diversity between countries and regions with high versus low burden of Alzheimer’s disease, using an alpha level of 0.05. Violin plots were used to visualize the distribution of alpha diversity of each sample within a country, region or group.

#### Compositional abundance

Bacterial species were grouped by phylum, and relative abundance was calculated for each phylum within a microbiome sample. The samples were again grouped by Alzheimer’s disease incidence (low and high) and visualized in a vertical bar plot to compare differences in relative abundance of each phylum between the groups. The abundance profile of each phylum was further visualized as a box plot with jittered data points, to highlight individual phylum differences between the groups.

#### Beta diversity

Beta diversity is a measure of diversity between different microbiome populations.^24^ This was used to assess differences in the overall community structure between Alzheimer’s disease groups. To assess beta diversity, a Bray–Curtis distance matrix was first calculated. This is based on microbial relative abundance counts and considers all species to produce a Bray–Curtis distance matrix that relates each sample to every other sample. Multidimensional scaling (MDS), otherwise known as principle coordinates analysis, was then applied to the Bray–Curtis distance matrix to visualize such distances in a 2D space. The data points represent samples, and are colour coded according to Alzheimer’s disease group. An increased distance between any two data points signifies increased dissimilarity between the two samples. Colour coding the data points by Alzheimer’s disease group helps to visualize how dissimilar the microbiome composition of the two groups are.

#### PERMANOVA

A permutational multivariate analysis of variance (PERMANOVA) was used to determine the top bacterial species that significantly differ in abundance between the high and low Alzheimer’s disease burden groups. This statistical test uses a multivariate extension of ANOVA, given the large number of unique species involved in analysis. It uses the Bray–Curtis distance matrix calculated above in beta diversity analyses and calculates whether the distance between samples from the different groups is larger than the distance between samples from the same group. The significance of this result is then tested by permuting the allocation of samples to groups and repeating the test, in this case over 999 permutations. If the ratio of between group distance to within group distance is consistently higher in the original data than the permutations, a statistically significant difference is observed. For this analysis, we used the adonis function in the R package *vegan.*^21^

#### Machine learning to predict Alzheimer’s disease burden from microbiome profile

The Statistical Inference of Associations between Microbial Composition And host phenoTypes (*SIAMCAT*) R package was used to predict the population burden of Alzheimer’s disease based on gut microbiome profile.^22^ It provides a robust statistical model to determine the relationship between the microbiome community composition and Alzheimer’s disease burden, whilst also exploring and accounting for confounding factors through an embedded function. This is especially important to consider in microbiome association studies due to the high risk of pseudo results. Other statistical modelling tools used include cross-validation, parameter selection and receiver operating characteristic (ROC) analysis.

To use the SIAMCAT pipeline, features with low overall abundance <1e^−04^ were filtered out of the dataset. The *check.associations()* function was used to calculate significance (*P* < 0.05) and metrics of association between microbial species and Alzheimer’s disease burden, using the non-parametric Wilcoxon test. Different effect sizes for the association were presented as AU-ROC and generalized Fold Change (gFC).

In order to train our model, we used a 10-times resampled 10-fold cross-validation scheme. This allows us to separate data into blocks, which can then be used to train the model with different block combinations. To evaluate how well the model performed, we used the *evaluate.predictions()* function. This produces an ROC and precision–recall (PR) curve, calculating the area under the curve (AUC) as a measure of model performance.

Confounder analysis was performed using the SIAMCAT package too. First, a conditional entropy check was performed to identify any interdependence of confounders on each other. It quantifies the unique information contained in one variable with respect to another, with a value of 0 highlighting identical nonsensical variables. Single covariate logistic regression analysis and Fisher tests were used to identify any significant correlation between the metadata variables age, sex and BMI grouping with the label Alzheimer’s disease burden. All three metadata variables were further investigated to check if they had confounding effects on individual microbial features. The SIAMCAT package does this by visualizing the variance explained by the label (in this case Alzheimer’s disease grouping) compared with the variance explained by each metadata variable.

### Statistical analysis

Statistical analyses were specific to each form of analysis. All statistical analyses used an alpha level of 0.05 and compared the groups of high and low Alzheimer’s disease burden separately for the Atlas and Curated dataset. The alpha diversity metric, Shannon index, was tested for statistical significance using the Wilcoxon signed rank test. The microbiome composition of the two groups was compared for statistical significance using PERMANOVA analysis, which is described in detail above. Finally, confounder analysis as part of the SIAMCAT machine learning package was tested for statistical significance using Fisher’s exact test.

## Results

**Figure 1:**
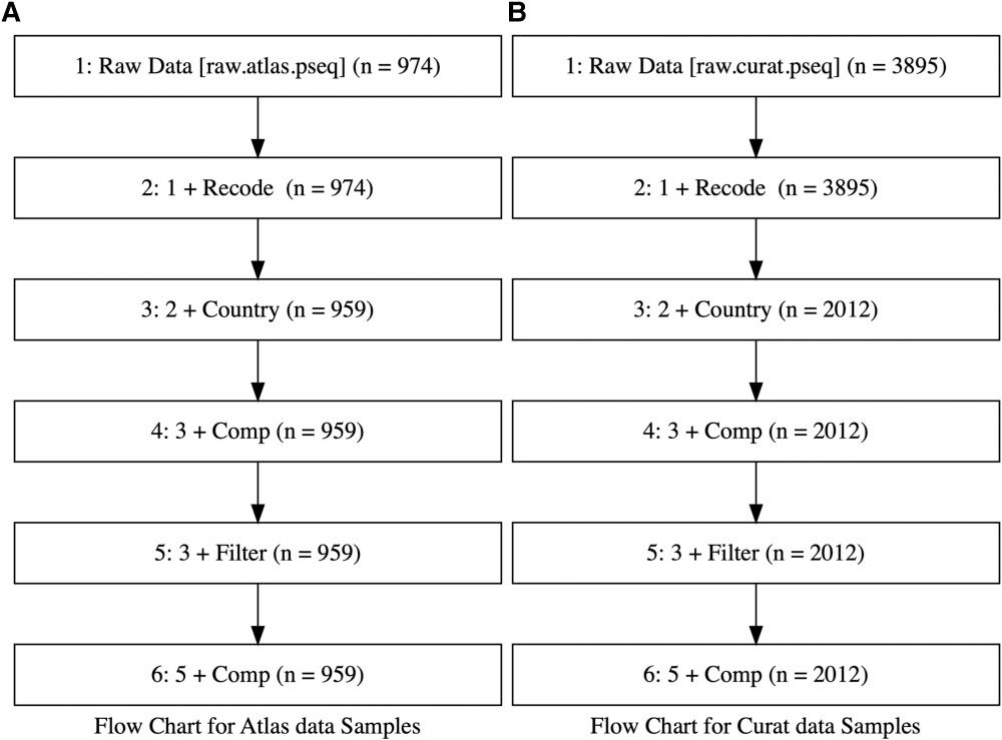
**Flow chart for (A) Atlas data samples and (B) Curated data samples, outlining the breakdown in sample numbers at each stage of filtering.** Atlas1006 baseline data included 974 samples, of which 959 samples (Mean age = 45) included geographical data—multi-nation geographical regions Scandinavia, Central Europe, South Europe, US and UKIE (UK and Ireland). The raw Curated dataset included 3895 samples, of which 2012 samples (Mean age = 42) were in adults from non-diseased states with relevant geographical data. Abbreviations: Comp, compositional (converting data to relative abundance).

Atlas1006 baseline data included 974 samples, of which 959 samples (Mean age = 45) were from healthy adults and included geographical data—multi-nation geographical regions Scandinavia, Central Europe, South Europe, US and UKIE (UK and Ireland) (Fig. 1). The raw Curated dataset included 3895 samples, of which 2012 samples (Mean age = 42) were from healthy adults with relevant geographical data (Fig. 1). The countries included were Netherlands, Denmark, Germany, Finland, France, Italy, Norway, Spain, Sweden, UK, US, Serbia, Belgium, Ireland, Austria, Luxembourg.

### Population parameters of Alzheimer’s disease

The countries and regions included were selected to match the regions and countries from the microbiome data: Scandinavia (Norway, Finland, Sweden), Central Europe (Netherlands, Belgium, Denmark, Germany), South Europe (Serbia, Italy, Spain, France), United States (US), UKIE (United Kingdom, Ireland), Luxembourg and Austria. DALY, prevalence and incidence rates (per 100 000 individuals) are demonstrated in Table 1, with mean values calculated for each region. Notably, mean incidence of Alzheimer’s disease was highest in Southern Europe, specifically in Serbia, and lowest in the US.

**Table 1:** Global burden of Alzheimer’s disease

Region	Country	DALY^a^	Prevalence^a^	Incidence^a^
Scandinavia				
	Norway	312.22	597.40	97.69
	Finland^b^	292.53	476.09	91.57
	Sweden	286.11	510.66	86.92
	Mean	296.96	528.05	92.06
Central Europe				
	Netherlands	326.97	583.81	92.95
	Denmark^b^	285.18	538.50	89.33
	Germany^c^	297.17	572.33	94.38
	Luxembourg	273.28	544.00	90.50
	Austria	294.73	591.01	97.21
	Belgium^c^	297.80	571.84	94.41
	Mean	295.86	566.91	93.13
South Europe				
	France	316.76	596.58	97.78
	Spain	313.83	607.60	98.84
	Italy	353.86	623.46	100.53
	Serbia	328.71	659.15	108.53
	Mean	328.29	621.70	101.42
US				
	United States	319.95	469.96	85.16
	Mean	319.95	469.96	85.16
UKIE				
	Ireland	302.26	572.10	94.33
	United Kingdom	293.85	545.73	92.26
	Mean	298.05	558.91	93.29

The countries and regions included were selected to match the regions and countries from the microbiome data: Scandinavia (Norway, Finland, Sweden), Central Europe (Netherlands, Belgium, Denmark, Germany), South Europe (Serbia, Italy, Spain, France), United States (US), UKIE (United Kingdom, Ireland), Luxembourg and Austria. DALY, prevalence and incidence rates (per 100 000 individuals) are demonstrated for each of these countries and regions, with mean values calculated for each region.

^a^Lancet 2016.

^b^Grouped by region according to Atlas dataset groupings.

^c^Present only in Curated dataset.

### Disease burden graphs

**Figure 2:**
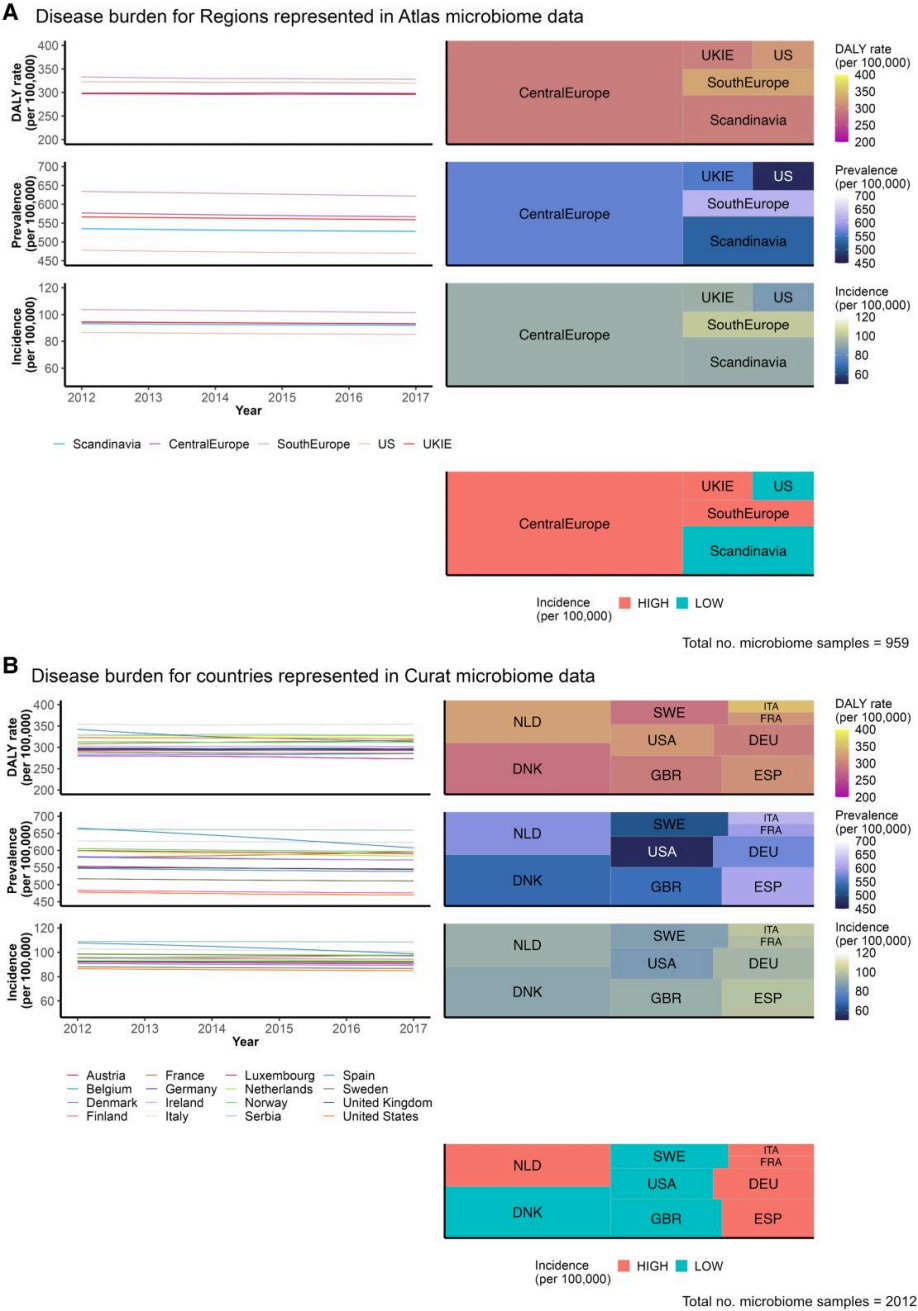
**Disease burden for (A) regions represented in Atlas microbiome data and (B) countries represented in Curated microbiome data.** The three metrics of disease burden investigated are DALY rate, prevalence and incidence, all per 100 000 individuals. The line graphs on the left show stability of each metric from 2012 to 2017. The heat maps on the right visualize these metrics by country. Finally, the tree map at the bottom of each figure part shows the split of countries or regions into groups of high and low Alzheimer’s disease burden using a threshold incidence of 92.5 per 100 000. Regions with a high incidence of Alzheimer’s disease were found to be Central Europe, South Europe and UKIE. Countries with a high incidence of Alzheimer’s disease include The Netherlands, Austria, Italy, France, Spain, Germany and Belgium. Abbreviations: DALY, disability-adjusted life year; DEU, Germany; DNK, Denmark; ESP, Spain; FRA, France; GBR, Great Britain; ITA, Italy; NLD, Netherlands; SWE, Sweden; UKIE, United Kingdom and Ireland, US, United States; USA, United States of America.

Using a threshold incidence of 92.5 per 100 000 to create approximately equal groups of countries and regions with high and low incidence of Alzheimer’s disease, regions with a high incidence of Alzheimer’s disease were found to be Central Europe, South Europe and UKIE. Countries with a high incidence of Alzheimer’s disease include the Netherlands, Austria, Italy, France, Spain, Germany and Belgium. This is visually demonstrated in both a heat map for each metric and as a tree map for the incidence metric in Fig. 2. In both maps, the area in tile represents the microbiome sample size. In the heat maps, the colour represents the DALY, prevalence or incidence rate, and in the tree map, the colour represents the final Alzheimer’s disease burden grouping. This figure further shows the stability of each metric from 2012 to 2017; a prudent point in ensuring that allocation to groups does not change depending on the year chosen.

### Demographic data

Host variables such as BMI, sex and age can all affect the microbiome and confound microbiome studies.^25^ Hence, demographic characteristics of the two disease groups were further investigated to identify confounders (Tables 2 and 3). In the Curated dataset (Table 3), the low disease group was proportionally composed of more female individuals, with higher BMI and higher age group. Similar findings were also seen in the Atlas dataset (Table 2), apart from BMI which was more similarly spread between the two disease groupings. Such potential confounders are further investigated for in the SIAMCAT analysis package, detailed below (Supplementary Fig. 4).

**Table 2:** Demographic data for Atlas dataset

Variable	Low disease group	High disease group	Total
Gender			
Female	129	412	541
Male	62	341	403
Unknown	15	0	15
Age			
Mean	42	45	45
SD	12	15	14
BMI			
Lean (BMI 18.5 + to 25)	97	340	437
Overweight (BMI 25 + to 30)	36	115	151
Obese (BMI 30 + to 35)	27	159	186
Morbid Obese (BMI 35 + to 40)	0	0	0
Severe Obese (BMI 40 + to 45)	0	0	0
Super Obese (BMI 45+)	0	0	0
Unknown	26	43	69

Host variables such as BMI, sex and age can all affect the microbiome and confound microbiome studies.^9^ Hence, demographic characteristics of the two disease groups were further investigated to identify confounders. In the Atlas dataset, the low disease group was proportionally composed of more female individuals, with higher age group. BMI was more similarly spread between the two disease groupings.

**Table 3:** Demographic data for Curated dataset

Variable	Low disease group	High disease group	Total
Gender			
Female	582	284	866
Male	201	239	440
Unknown	311	395	706
Age			
Mean	48	34	42
SD	16	16	17
BMI			
Lean (BMI 18.5 + to 25)	213	397	610
Overweight (BMI 25 + to 30)	218	110	328
Obese (BMI 30 + to 35)	172	18	190
Morbid obese (BMI 35 + to 40)	11	0	11
Severe obese (BMI 40 + to 45)	34	2	36
Super obese (BMI 45+)	4	12	16
Unknown	442	379	821

Host variables such as BMI, sex and age can all affect the microbiome and confound microbiome studies.^9^ Hence, demographic characteristics of the two disease groups were further investigated to identify confounders. In the Curated dataset, the low disease group was proportionally composed of more female individuals, with higher BMI and higher age group.

### Alpha diversity

**Figure 3:**
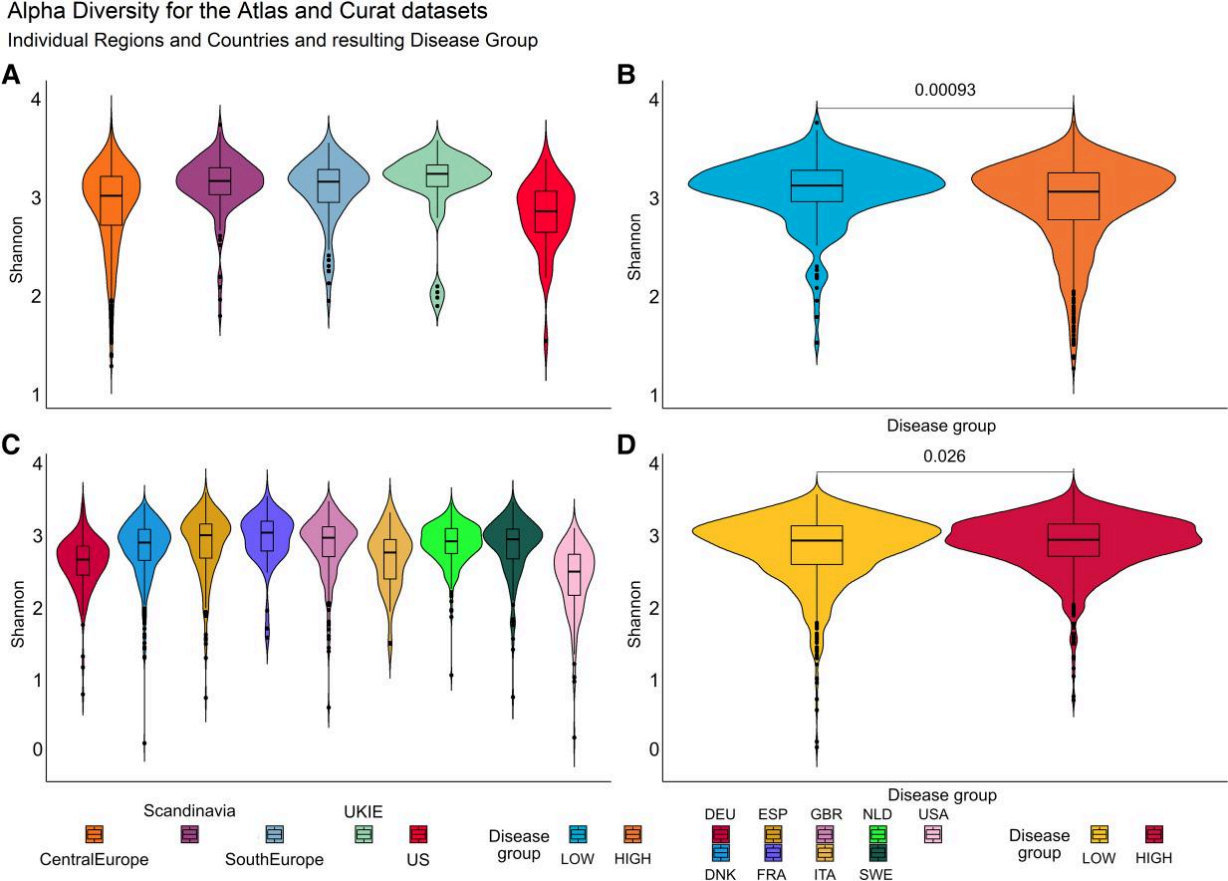
**Alpha diversity violin plots for the Atlas (A, B) and Curated (C, D) datasets.** Alpha diversity was quantified using the Shannon index, given its sensitivity to both species richness and evenness. The countries and regions were grouped into high (countries = Netherlands, Austria, Italy, France, Spain, Germany and Belgium; regions = Central Europe, South Europe, UKIE) and low (countries = UK, USA, Denmark, Sweden; regions = Scandinavia, US) incidence of Alzheimer’s disease. The Wilcoxon signed rank test was used to assess for differences in alpha diversity between the two groups with high versus low burden of Alzheimer’s disease, using an alpha level of 0.05, for both regions (**B**) and countries (**D**). Violin plots were used to visualize the distribution of alpha diversity of each sample within a region (**A**), country (**C**) or group (**B, D**). The plots demonstrate that in regions and countries with a high incidence of Alzheimer’s disease, there is a significantly lower diversity of the gut microbiome for both datasets (Atlas: *P* < 0.001, Curated: *P* = 0.026). Abbreviations: DEU, Germany; DNK, Denmark; ESP, Spain; FRA, France; GBR, Great Britain; ITA, Italy; NLD, Netherlands; SWE, Sweden; UKIE, United Kingdom and Ireland, US, United States; USA, United States of America.

Alpha diversity is a measure of within sample species richness and evenness and was quantified here using the Shannon index.^23^ The Wilcoxon signed rank test was used to assess for differences in alpha diversity between countries and regions with high versus low burden of Alzheimer’s disease, using an alpha level of 0.05. Violin plots were used to visualize the distribution of alpha diversity of each sample within a country, region or group. We find that in regions and countries with a high incidence of Alzheimer’s disease, there is a significantly lower diversity of the gut microbiome (Atlas: *P* < 0.001, Curated: *P* = 0.026) (Fig. 3). Further, the US was identified as having particularly lower alpha diversity in both datasets, compared with other regions and countries.

### Abundance graph

**Figure 4:**
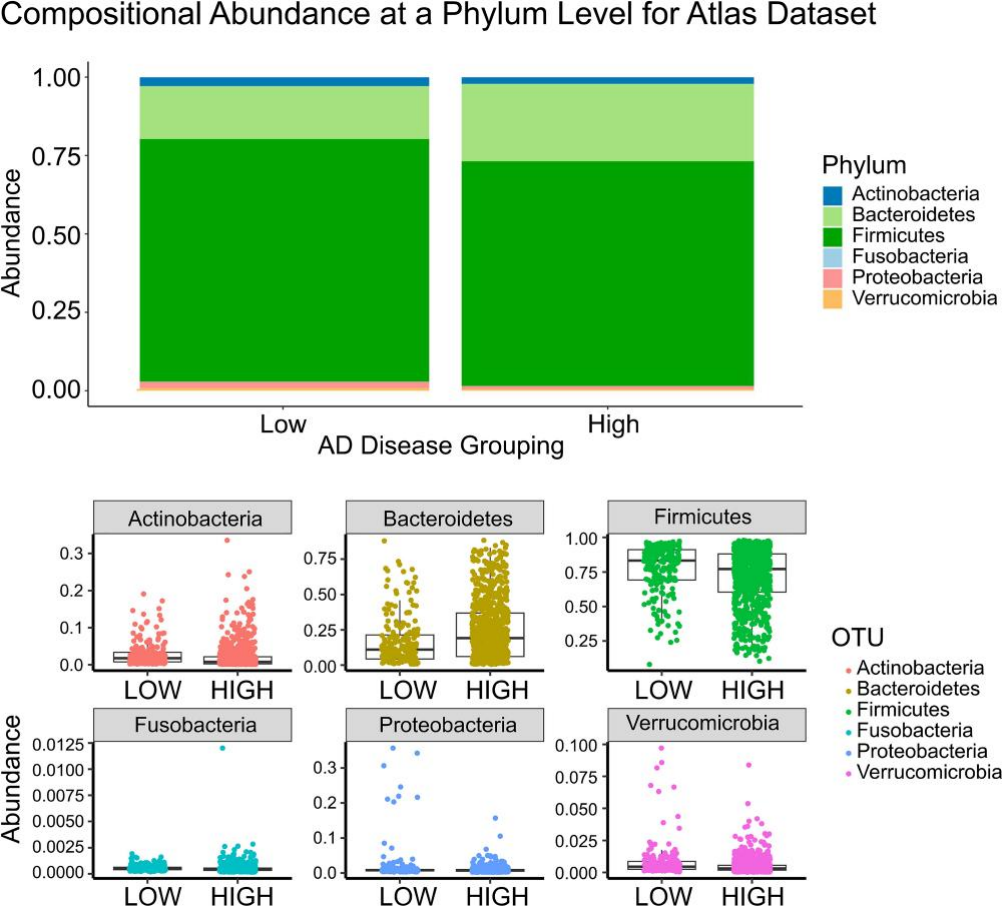
**Abundance in the disease grouping in Atlas microbiome dataset.** Bacterial species were grouped by phylum, and relative abundance was calculated for each phylum within a microbiome sample. The samples were grouped by Alzheimer’s disease incidence (low and high) and visualized in a vertical bar plot to compare differences in relative abundance of each phylum between the groups. The abundance profile of each phylum was further visualized as a box plot with jittered data points, to highlight individual phylum differences between the groups. Examining abundances at the phyla level visually revealed no reproducible differences between the two datasets. Differential abundance is statistically assessed further in Fig. 7.

**Figure 5:**
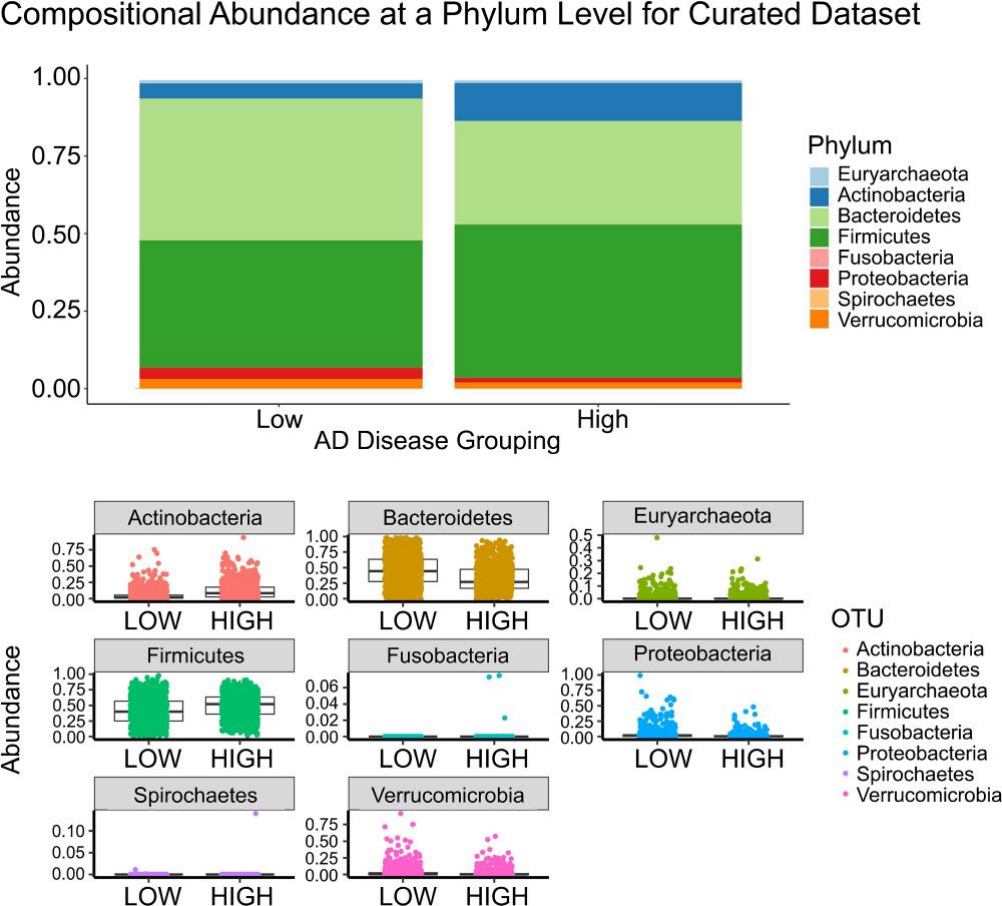
**Abundance in the disease grouping in Curated microbiome dataset.** Bacterial species were grouped by phylum, and relative abundance was calculated for each phylum within a microbiome sample. The samples were grouped by Alzheimer’s disease incidence (low and high) and visualized in a vertical bar plot to compare differences in relative abundance of each phylum between the groups. The abundance profile of each phylum was further visualized as a box plot with jittered data points, to highlight individual phylum differences between the groups. Examining abundances at the phyla level visually revealed no reproducible differences between the two datasets. Differential abundance is statistically assessed further in Fig. 7.

The samples were grouped by Alzheimer’s disease incidence (low and high) and visualized in a vertical bar plot to compare differences in relative abundance of each phylum between the groups (Fig. 4: Atlas Dataset, Fig. 5: Curated Dataset). The abundance profile of each phylum was further visualized as a box plot with jittered data points, to highlight individual phylum differences between the groups (Fig. 4: Atlas Dataset, Fig. 5: Curated Dataset). Examining abundances at the phylum level revealed no reproducible differences for any phylum between the two datasets (Figs 4 and 5). This reinforces the view that species and even strain level functional differences may exist that contribute to disease pathogenesis.^26^

### Beta diversity

**Figure 6:**
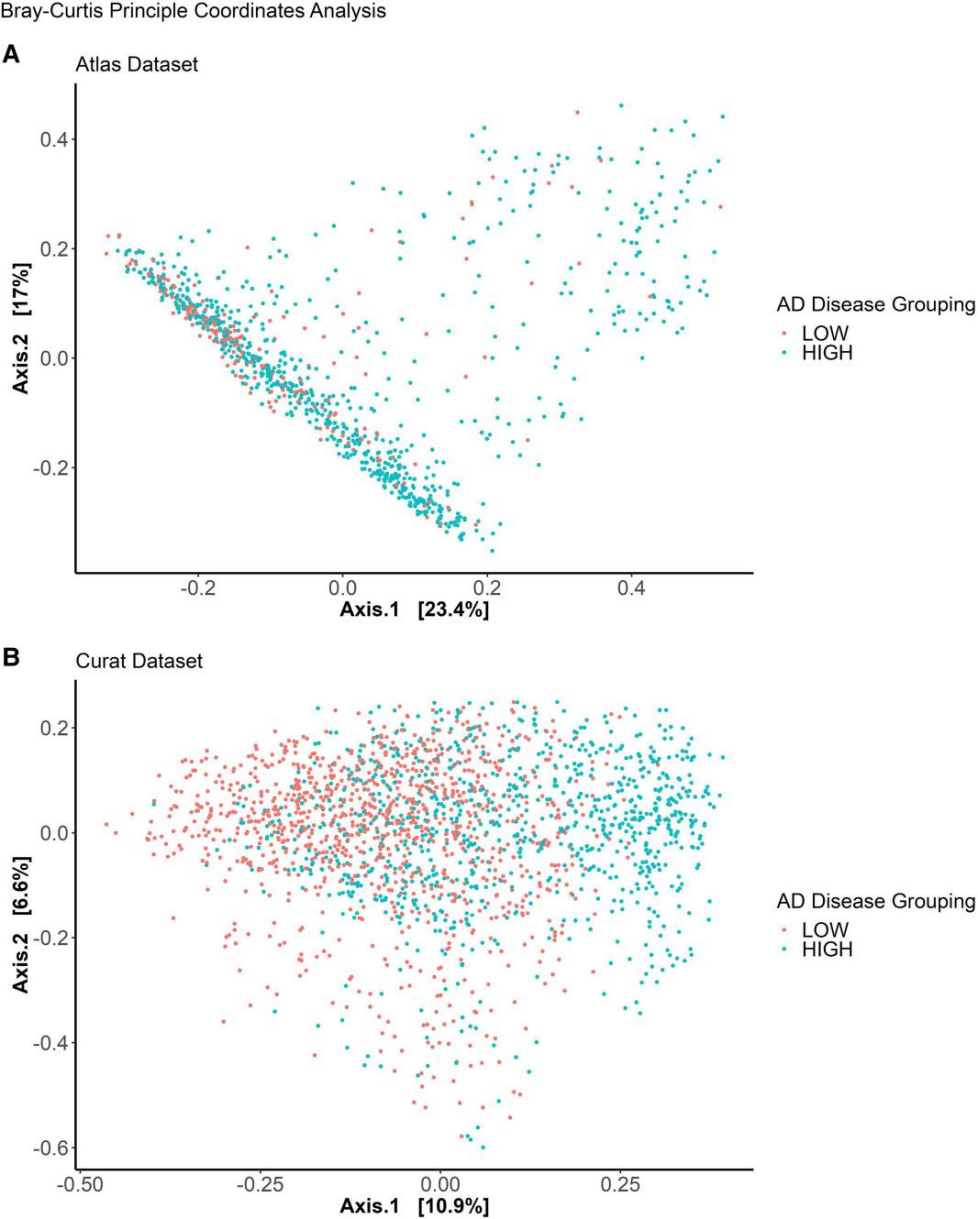
**Bray–Curtis principal coordinates analysis showing beta diversity of microbiome samples in countries and regions with low and high incidence of Alzheimer’s disease for Atlas dataset (A) and Curated dataset (B).** Beta diversity visualizes the dissimilarity in composition between microbiome samples for both Alzheimer’s disease groups. Each point on the graphs represents a sample. The distance between points are representative of their similarity in microbiome composition, with closer distance signifying more similar microbiome composition. While the distribution of samples between Alzheimer’s disease groups differs, all samples are grouped within the same overall region. This is further tested for statistical significance using the PERMANOVA test, outlined in Fig. 7.

Beta diversity is shown in Fig. 6 and visualizes the dissimilarity in composition between microbiome samples for both Alzheimer’s disease groups.^24^ Each point on the graphs represents a sample. The distance between points are representative of their similarity in microbiome composition, with closer distance signifying more similar microbiome composition. The distribution of samples between the Alzheimer’s disease groups differs, although no clear clustering is seen of the groups across both datasets.

### PERMANOVA

**Figure 7:**
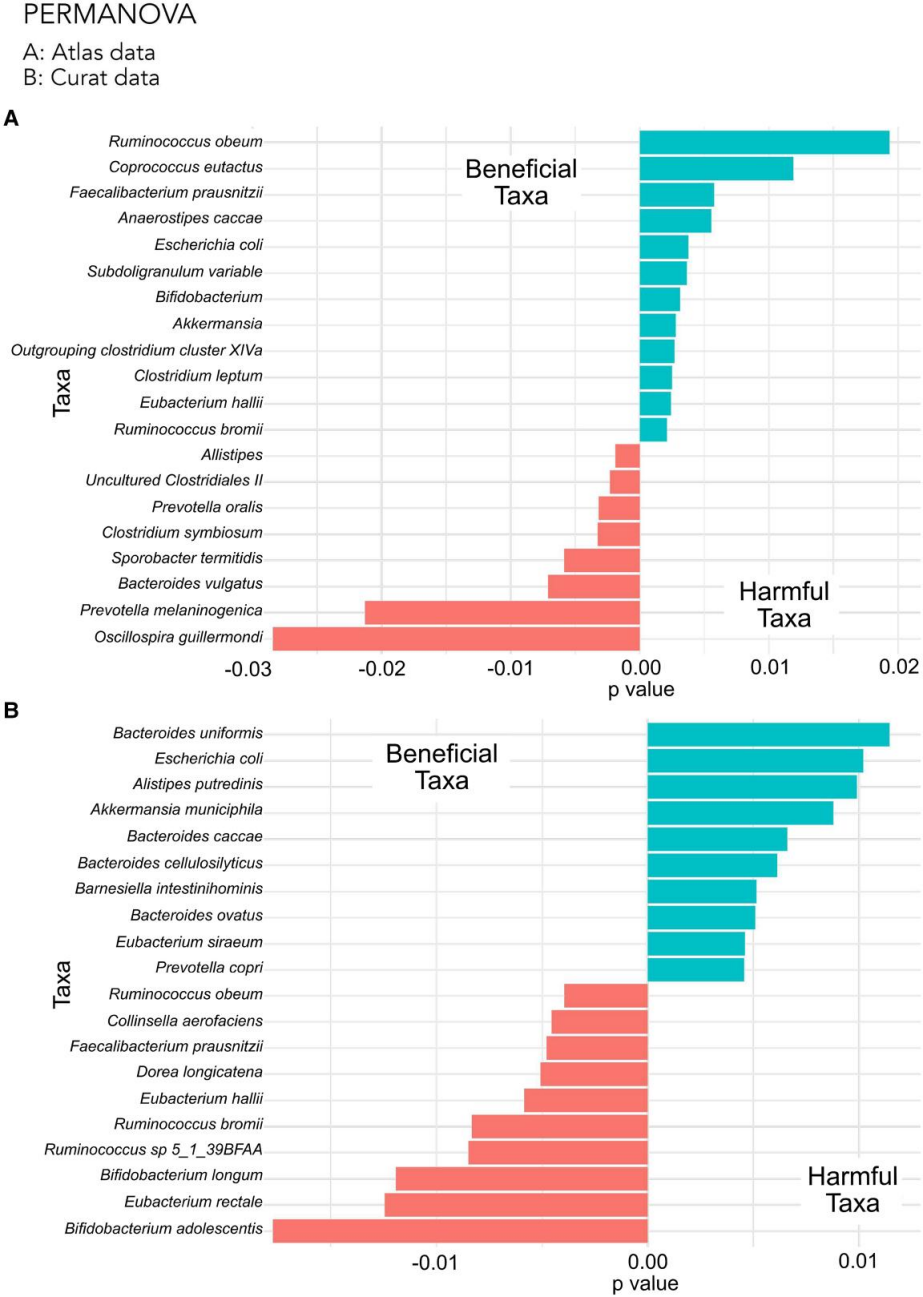
**PERMANOVA results identifying significant differences between several taxa in countries with high versus low incidence of Alzheimer’s disease for Atlas dataset (A) and Curated dataset (B).** A PERMANOVA was used to determine the top bacterial species that significantly differ in abundance between the high and low Alzheimer’s disease burden groups. It tests the significance of the relationship between Alzheimer’s disease burden group and composition of the microbiome, using the adonis function in the R package *vegan.*^5^ The Bray–Curtis distance matrix calculated in beta diversity analyses was used to quantitatively measure the dissimilarity between the microbiome communities of high and low Alzheimer’s disease burden groups, and tested for statistical significance by permuting the allocation of samples to groups 999 times. This revealed significant differences between several taxa in countries with high versus low incidence of Alzheimer’s disease (*P* < 0.05).

A PERMANOVA was thus performed to test for significant differences between the groups. The Bray–Curtis distance matrix calculated in beta diversity analyses was used here to quantitatively measure the dissimilarity between the microbiome communities of high and low Alzheimer’s disease burden groups, and tested for statistical significance by permuting the allocation of samples to groups 999 times. The PERMANOVA test revealed significant differences between the two groups (*P* < 0.001). It further identified several taxa with contributed most to the differences seen between the two Alzheimer’s disease burden groups (Fig. 7). *Escherichia coli* and *Akkermansia sp.* were consistently identified in both datasets to be associated with low Alzheimer’s disease burden (Supplementary Table 1). No species were consistently associated with high Alzheimer’s disease burden across both datasets.

### SIAMCAT

The *SIAMCAT* package was used to identify differentially abundant features between the two disease groupings in both datasets.^22^ Reproducible taxa differences between the two datasets were seen with *Clostridium sp., Haemophilus sp.* and *Escherichia coli*; all of which were associated with low Alzheimer’s disease grouping suggesting a protective element to these bacteria (Supplementary Fig. 1, A: Atlas Dataset, B: Curated Dataset).

Finally, using a supervised learning model, we can predict the incidence of Alzheimer’s disease within a region or country based on the microbiome profile (Supplementary Fig. 2, A: Atlas Dataset, B: Curated Dataset). The graphs shown in Supplementary Fig. 2 plot the true positive rate against the false positive rate, with a higher AUC meaning better model fit.

They show that the model has high predictive value for both datasets with Area under the receiver operating characteristic (AU-ROC) values of 0.889 and 0.927 for the Atlas and Curated datasets, respectively.

### Model interpretation plots

Model interpretation plots were used to identify which metagenomic features had the highest predictive value for the burden of Alzheimer’s disease (Supplementary Fig. 3, A: Atlas Dataset, B: Curated Dataset). Notably, consistent taxonomic features between the two datasets include *Bacillus sp.* as a strong predictive factor for countries and regions with a high burden of Alzheimer’s disease, and *Haemophilus sp.* as a strong predictive factor for countries and regions with a low burden of Alzheimer’s disease.

### Confounder analysis

The SIAMCAT package was used to analyse the metadata variables age, gender and BMI for any potential confounding (Supplementary Fig. 4, A: Atlas Dataset, B: Curated Dataset). A conditional entropy check was first performed to identify any interdependence of confounders on each other or the variable of interest, Alzheimer’s disease burden. It quantifies the unique information contained in one variable with respect to another, with a value of 0 highlighting identical nonsensical variables, none of which were identified here for both datasets.

Single covariate logistic regression analysis and Fisher tests were used to identify any correlation between the metadata variables age, sex and BMI grouping with the label Alzheimer’s disease burden. These analyses show that the male sex was correlated with increased Alzheimer’s disease burden in both datasets with a significant Fisher test *P*-value in both datasets (*P* < 0.001). However, age and BMI had opposite findings in the two datasets, both with significant correlation (Fisher test *P* < 0.001).

All three metadata variables were further investigated to check if they had confounding effects on individual microbial features. The SIAMCAT package does this by visualizing the variance explained by the label (in this case Alzheimer’s disease grouping) compared with the variance explained by each metadata variable. More variance in microbial features was explained by Alzheimer’s disease grouping than each metadata variable, confirming that these metadata variables were not confounding label associations.

## Discussion

The interactions between the gut microbiome and the brain are bidirectional, commonly referred to as the gut-brain axis. The interactions are 3-fold, involving neural, endocrine and immunological pathways, integrated with the sympathetic and parasympathetic arms of the autonomic nervous system and the enteric nervous system.^27^ The field is rapidly developing with increasing evidence for each of these pathways: BDNF mRNA is upregulated in the dentate gyrus of the hippocampus of germ-free animals; increased levels of the stress hormone, corticosterone, are noted in the plasma of germ-free mice; and down-regulation of NMDA and 5-HT1A receptors were noted in germ-free mice.^28^

Our work has endeavoured to further strengthen the link between the gut microbiome and the brain, specifically looking at its links with the most common neurodegenerative condition, Alzheimer’s disease.

Our results show three key findings:

1. Countries and regions with a high incidence of Alzheimer’s disease show lower microbiome diversity.
2. Differentially abundant taxa have been identified between countries and regions with a high versus low incidence of Alzheimer’s disease.
3. Machine learning can predict the incidence of Alzheimer’s disease based on population microbiome profile.

### Microbiome diversity

The gut microbiome of patients with Alzheimer’s disease has shown decreased microbial diversity in previous studies.^14,29^ We confirm this finding in our study in two different datasets.

Coevolution between microbial communities and their hosts plays a role in promoting alpha diversity. A 2-fold natural selection process has been hypothesized, with top-down pressure from host to microbial community to favour a stable society with a high degree of functional redundancy, and bottom-up selection pressure from microbial cells to become functionally specialized.^30^ Within the microbiome, both synergistic and antagonistic relationships exist, in the form of direct competition for limited resources, and conversely, positive feedback loops whereby the product of one microbe becomes the substrate for another, respectively.

The implications of reduced microbial diversity are vast, and include neural, endocrine and immune consequences.

From a neural perspective, gut microbes have been identified to communicate with the CNS through the direct production of neurotransmitters, such as GABA, serotonin and histamine, as well as indirectly through intermediary molecules such as short chain fatty acids (SCFAs), tryptophan and secondary bile acids.^31-35^ It is likely that a reduced gut microbial diversity would impact the communication within the gut-brain axis, either beneficially or harmfully.

From an endocrine perspective, gut microbial-derived genes and pathways have been shown to be involved in the metabolism of endogenous steroid hormones and endocrine disrupting chemicals.^36^ Further, the production of gut-derived hormones from enteroendocrine cells has been shown to be influenced by gut microbiota.^34^ Thus, a state of reduced microbial diversity can influence the endogenous hormonal state of the host positively or negatively depending on which species have proliferated in abundance. Further, the amount of calories available to the host from food is modulated to a significant degree by the gut microbiota.^37^ Calorie-plus conditions, such as diabetes mellitus, can promote microglial activation in Alzheimer’s disease mice, supporting the role of an inflammatory process as a link between Alzheimer’s disease and T2DM.^38^

From an immune perspective, dysbiosis is correlated with increased levels of proinflammatory cytokine production by immune cells, such as TNF-alpha.^39^ Microbial-derived SCFA levels are reduced in states of lower microbial diversity, as communication between gut microbiota supports their production through product-substrate utilization, such as in the case of *Bacteroides thetaiotaomicron* producing acetate that is utilized by *Eubacterium hallii* to generate butyrate.^40,41^ Such SCFAs have important roles in gut-immune-brain crosstalk, through influences on regulatory T cell generation and homeostasis, binding to G-protein-coupled receptors with downstream stimulation of secretion of glucagon-like peptide 1 and peptide YY, and influences on blood brain barrier permeability,^40,42-45^ Thus, a dysbiotic microbiome could hypothetically become a proinflammatory microbiome, with reduced intestinal barrier function and release of proinflammatory cytokines, leading to an inflammatory process that could extend to the brain and its microglia.

### Taxa differences

Phyla level differences are contradictory for Atlas and Curated datasets, highlighting the fact that each phylum is composed of multiple different taxa which can have very different effects on the microbiome, its diversity and the rest of the body. It is thus a futile exercise to model the microbiome based on phyla alone. Individual taxa differences must be further characterized, in order to better model the complex microbiome network. Reproducible taxa differences between the two datasets are highlighted in Supplementary Table 1, as confirmed by PERMANOVA and SIAMCAT analysis.

No taxa were consistently associated with regions with high Alzheimer’s disease burden between the two datasets for both PERMANOVA and SIAMCAT analysis. Reproducible findings were however seen in association with regions with low burden of Alzheimer’s disease: *Escherichia coli (E. coli), Akkermansia sp.* and *Haemophilus sp*.

*Escherichia coli* was interestingly associated with low Alzheimer’s disease burden in both PERMANOVA and SIAMCAT analysis. Previous studies show conflicting data with one group demonstrating a higher abundance, and another showing lower abundance of *Escherichia sp.* in amyloid-beta positive patients.^46^ Strain-level differences may explain the conflicting findings at a species level here.

With regards to *Akkermansia* species, a substantial amount of evidence exists supporting a protective role in Alzheimer’s disease. Potential mechanisms include: downregulation of proinflammatory cytokines, TNF-alpha and interferon-gamma, in the colon^47^; and production of SCFAs such as acetate and propionate.^48^ Although our study demonstrates correlation on a population level, causation has been established in APP/PS1 mouse models; these were fed with *Akkermansia municiphila* and subsequently developed reduced amyloid-beta 40–42 levels in CSF, and improved completion rate in Y maze tests.^49^ Our finding here is thus well supported by the literature, which also underscores the validity of a population-based approach as used in this study.

The significance of SCFA production is an area warranting further exploration. Acetate, proprionate and butyrate seem to be protective as supported by a recent study by Ling’s group, where they found a reduction in butyrate producing bacteria such as *Faecalibacterium* and increase in lactate producing bacteria such as *Bifidobacterium* in Alzheimer’s disease microbiome, which were both significantly correlated with host pro- and anti-inflammatory cytokines as well as clinical indicators of Alzheimer’s disease in the host.^50^

Finally, the literature on *Haemophilus* species is sparing, but a recent study comparing Alzheimer’s disease patients with healthy seniors confirmed our findings of reduced *Haemophilus* abundance in Alzheimer’s disease patients.^51^

What can be concluded from the above is that reproducibility must be established in multiple studies with large sample sizes to prove substantive correlation, prior to initiating studies to establish function and causation such as in the case of *Akkermansia* species. Further, strain-level analyses may be required given conflicting data that have arisen in previous studies and here too at a species level.

### Machine learning

The SIAMCAT R package successfully distinguishes between cases and controls given a sufficiently large dataset of at least 100 samples, as is the case with our sample size of 959 and 2012 for Atlas and Curated datasets, respectively.^22^ Furthermore, the SIAMCAT package has been shown to extract accurate microbial signatures where there is a lack of separation in ordination analysis, as is the case in both of our datasets. As a result, we have shown that machine learning can be used to predict Alzheimer’s disease incidence based on population microbiome profile with significant AU-ROC values of 0.889 and 0.927, respectively; supporting the hypothesis that the microbiome should be modelled as a network rather than individual taxa with individual roles.

Such network interactions are vast, and have even led to the development of specific computational tools to map the microbial ‘interactome’.^52^ Examples of such interactions include metabolite transfer, substrate competition, horizontal gene transfer and predator–prey interactions when considering viruses and fungi too.^53-56^ Further analyses should work to identify the interactions underlying the model network characterized through the SIAMCAT package and how exactly they influence the pathogenesis of Alzheimer’s disease.

## Conclusion

To conclude, our study suggests a significant role of the human gut microbiome in the pathology of Alzheimer’s disease at a population level. Neuroinflammation has been shown to play a significant role in neurodegeneration^57^; and Alzheimer’s disease brain has been shown to suffer neuroinflammatory changes, including increased abundance of disease-associated astrocytes and microglia,^58^ as well as increased expression of inflammatory cytokines.^59^ We have illustrated increasing evidence to suggest a role of the gut microbiome in this inflammatory process, and future studies will need to characterize the pathways of pathogenesis involved, and thus translationally pave the way for therapeutic targets.

## Research in context

### Evidence before this study

We searched PubMed for studies published in English using the following search teams: (human gut microbiome[MeSH Terms]) AND (Alzheimer's[MeSH Terms]). Although there are studies and meta-analyses looking at differences in the gut microbiota between patients with Alzheimer’s disease and controls, these are of relatively small sample size, ranging from 17 to 800 subjects. Sample size is particularly important in microbiome studies, where the number of different bacteria is vast, the effect sizes are potentially small, and thus the risk of incorrect statistical significance of associations is high. Furthermore, they do not employ machine learning methods to adjust for confounders, especially unidentifiable confounders not currently understood in the literature.

### Added value of this study

Here we analyse correlations between the gut microbiota of healthy and Alzheimer’s disease patients, with a much larger sample size of almost 4000, thus increasing the power of the study. We identify reduced microbiome diversity in Alzheimer’s disease patients, and furthermore can predict the population incidence of Alzheimer’s disease using the country’s gut microbiota profile. We thus provide stronger evidence of a link, and also identify bacterial taxa that consistently correlate with risk of Alzheimer’s disease at larger sample numbers.

### Implications of all the available evidence

Our findings support the hypothesis that increased risk of Alzheimer’s disease may be precipitated by gut microbiota differences, whether that is on an individual bacterial abundance level, on an overall microbiome diversity level, or through another as yet unidentified mechanism. Further research needs to be undertaken to elucidate the underlying mechanism, and thus pave the way for microbiome-derived therapeutics.

## Supplementary Material

fcaf059_Supplementary_Data

## Data Availability

De-identified patient microbiome data were obtained from two large, open-access microbiome data sets, referred to as Atlas (*n* = 959)^15^ and Curated (*n* = 2012).^16^ Data on the burden of Alzheimer’s disease were obtained from the Global Burden of Disease database for the years 2012 to 2017 inclusive.^18^ All datasets are publicly available. The R markdown script, including all data analysis, is also currently available on GitHub (via thesharmalab.com).
