# Supplementary material for: Machine learning determines the incidence of Alzheimer’s disease based on population gut microbiome profile
Source: Brain Commun. 2025 Apr 15;7(2):fcaf059. doi: 10.1093/braincomms/fcaf059 (PMC11999016; doi:10.1093/braincomms/fcaf059)
Supplement: fcaf059_Supplementary_Data [file fcaf059_supplementary_data.pdf]

Supplementary Table 1

Reproducible taxa differences between regions and countries with varying burden of AD in the Atlas and Curated datasets. No taxa were consistently associated with regions with high AD burden between the two datasets for both PERMANOVA and SIAMCAT analysis. Reproducible findings were however seen in association with regions with low burden of AD: *Escherichia coli (E. coli), Akkermansia sp.,* and *Haemophilus sp*.

	Taxa associated with high AD burden	Taxa associated with low AD burden (Phyla)
PERMANOVA		*Escherichia coli* (Proteobacteria)
		Akkermansia sp. (Verrucomicrobia)
SIAMCAT		*Haemophilus sp.* (Proteobacteria)
		Escherichia coli (Proteobacteria)

Supplementary Figure 1

Differentially abundant features showing top 40 features as produced by SIAMCAT package for Atlas dataset (A) and Curated dataset (B). The SIAMCAT package was used to identify differentially abundant features between the two disease groupings in both datasets. Reproducible taxa differences between the two datasets were seen with *Clostridium sp., Haemophilus sp.* and *Escherichia coli*; all of which were associated with low AD disease grouping.

Supplementary Figure 1

A

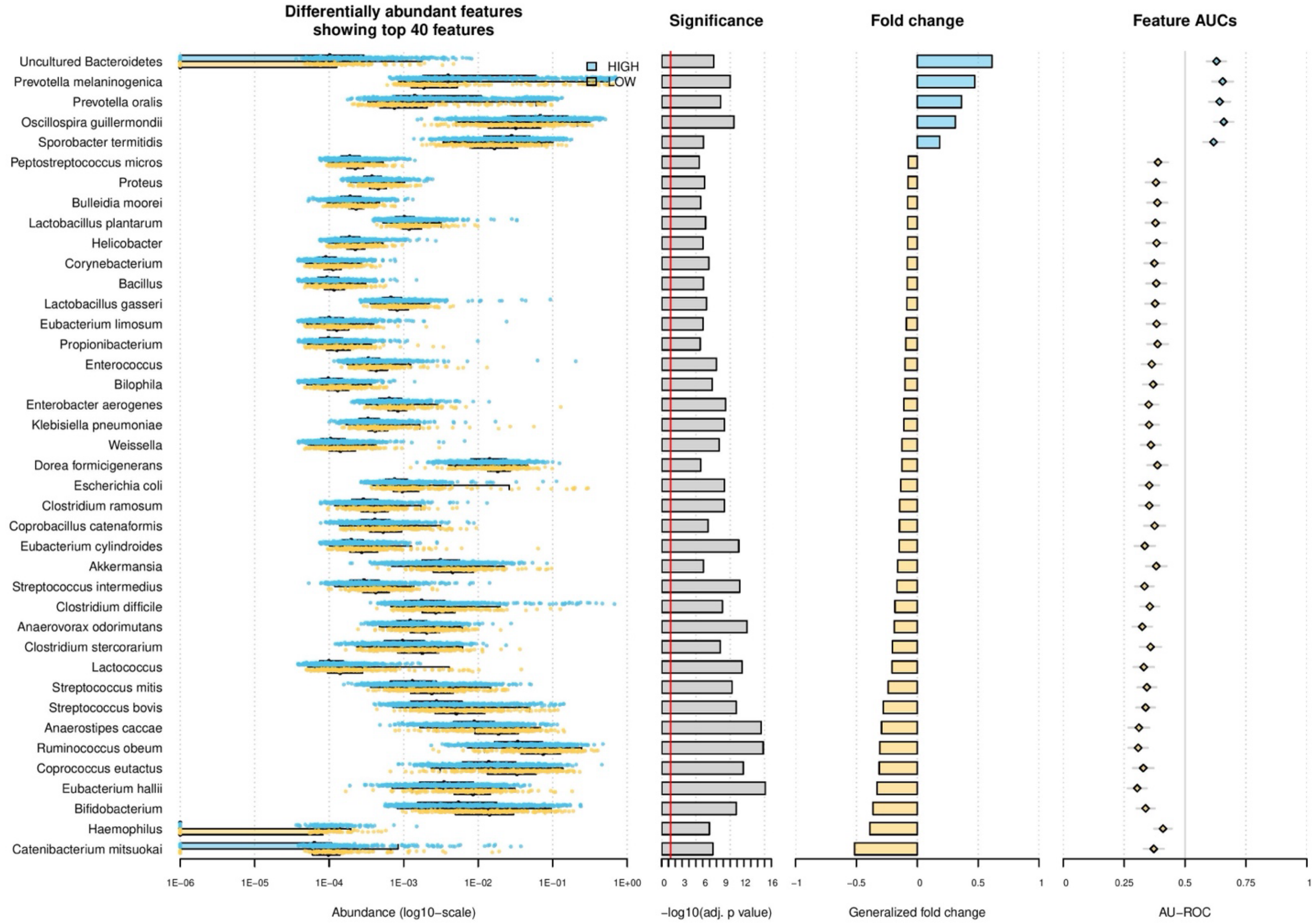

Supplementary Figure 1

B

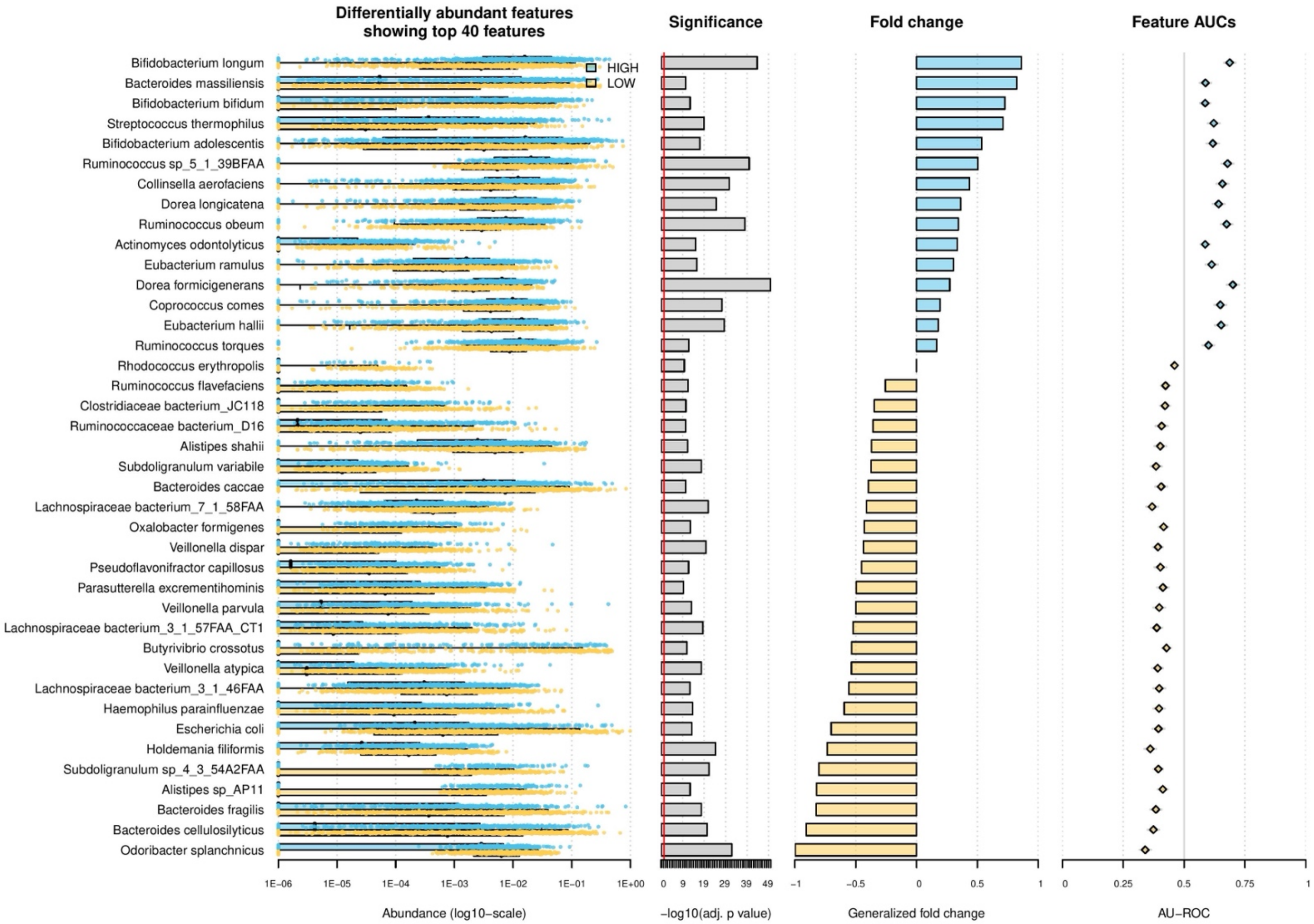

Supplementary Figure 2

Receiver operating characteristic (ROC) and precision-recall curves for the model predicting AD burden based on microbiome profile for Atlas dataset (A) and Curated dataset (B). This uses a supervised learning model to predict the incidence of AD within a region or country based on the microbiome profile. The graphs shown plot the true positive rate against the false positive rate, with a higher area under the curve meaning better model fit. They show that the model has high predictive value for both datasets with Area under the receiver operating characteristic (AUROC) values of 0·889 and 0·927 for the Atlas and Curated datasets respectively.

A

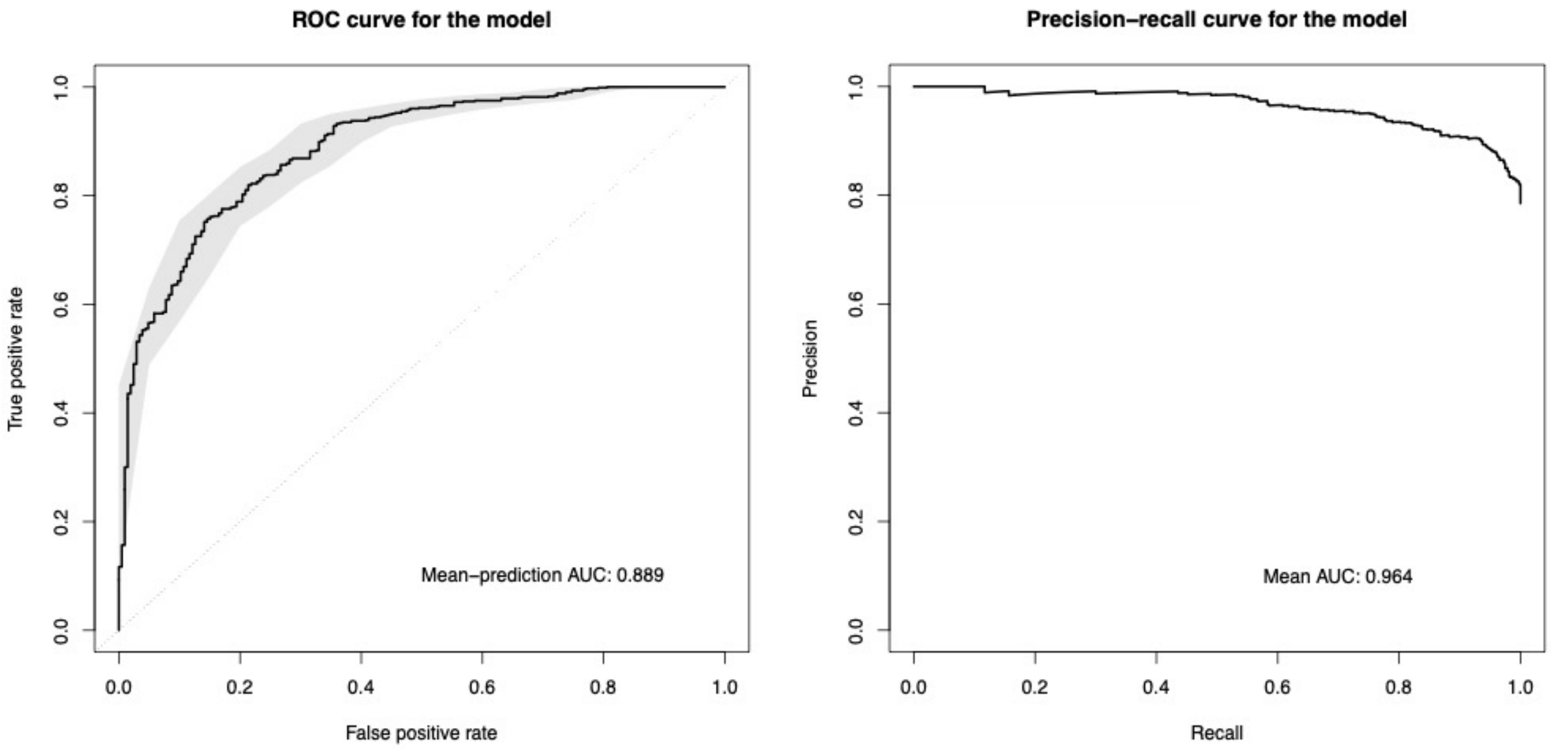

Supplementary Figure 2

B

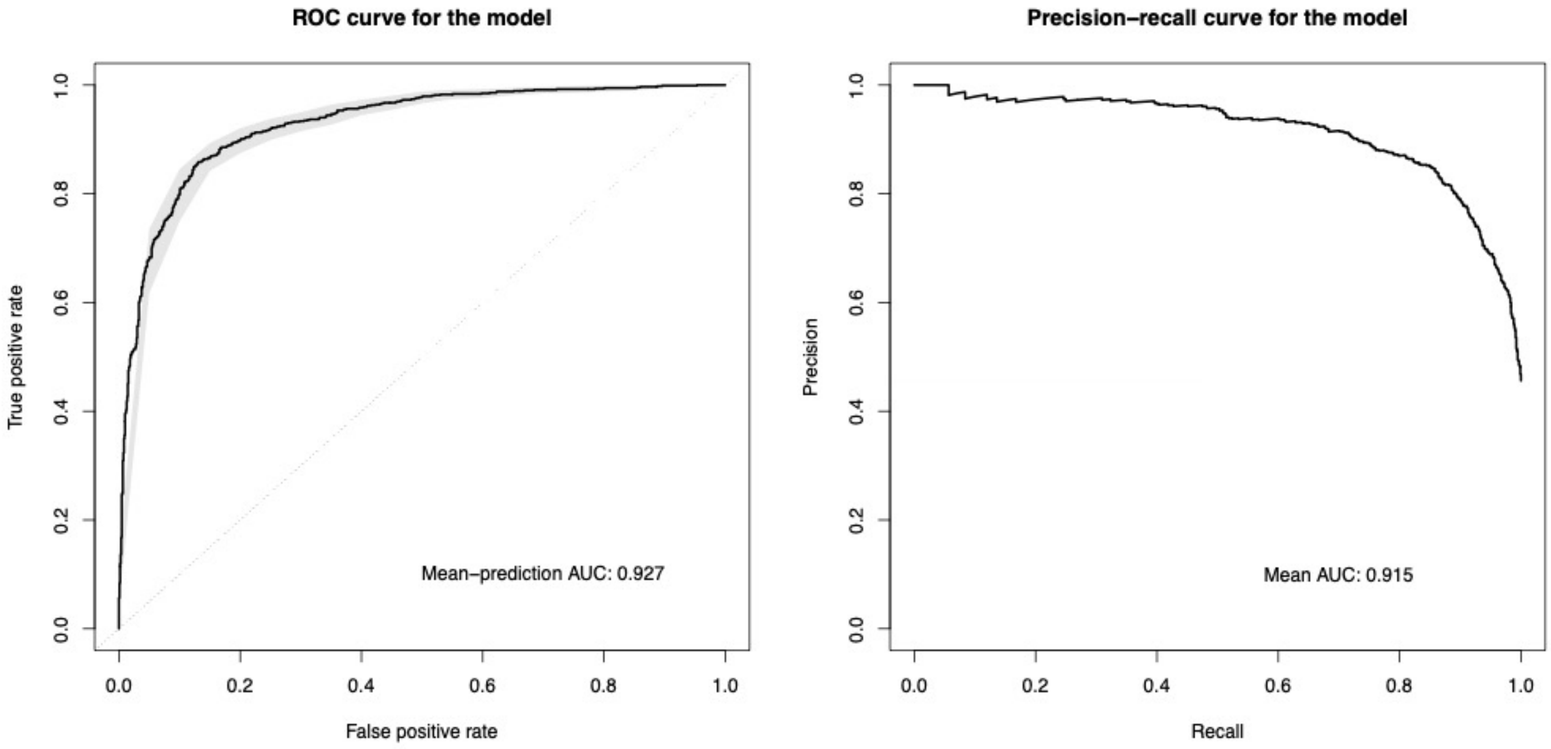

Supplementary Figure 3

Model interpretation plots of metagenomic features with predictive value for the burden of AD for Atlas Dataset (A) and Curated Dataset (B). Model interpretation plots were used to identify which metagenomic features had the highest predictive value for the burden of AD. Notably, consistent taxonomic features between the two datasets include *Bacillus sp.* as a strong predictive factor for countries and regions with a high burden of AD, and *Haemophilus sp.* as a strong predictive factor for countries and regions with a low burden of AD.

Supplementary Figure 3

A

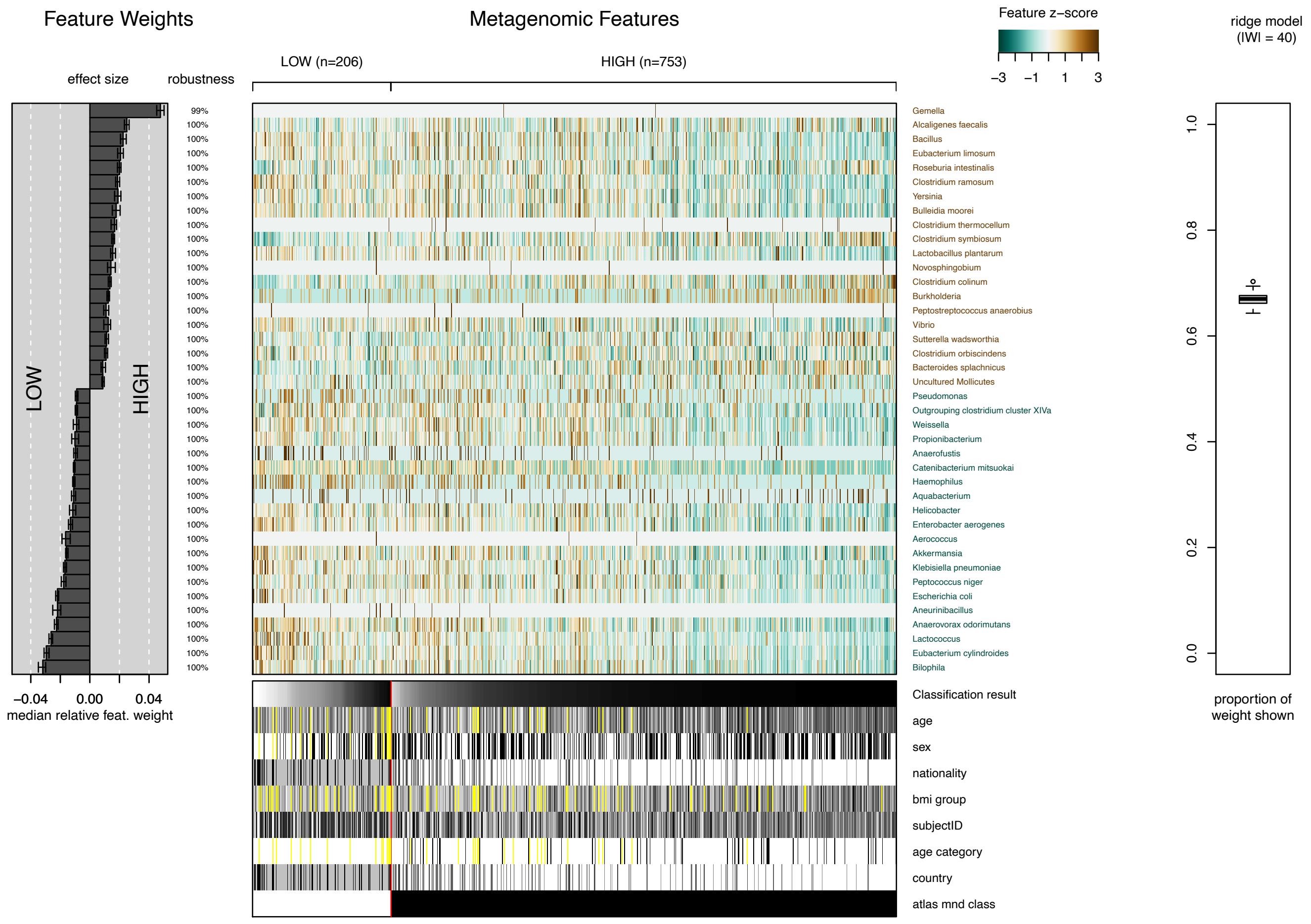

Supplementary Figure 3

B

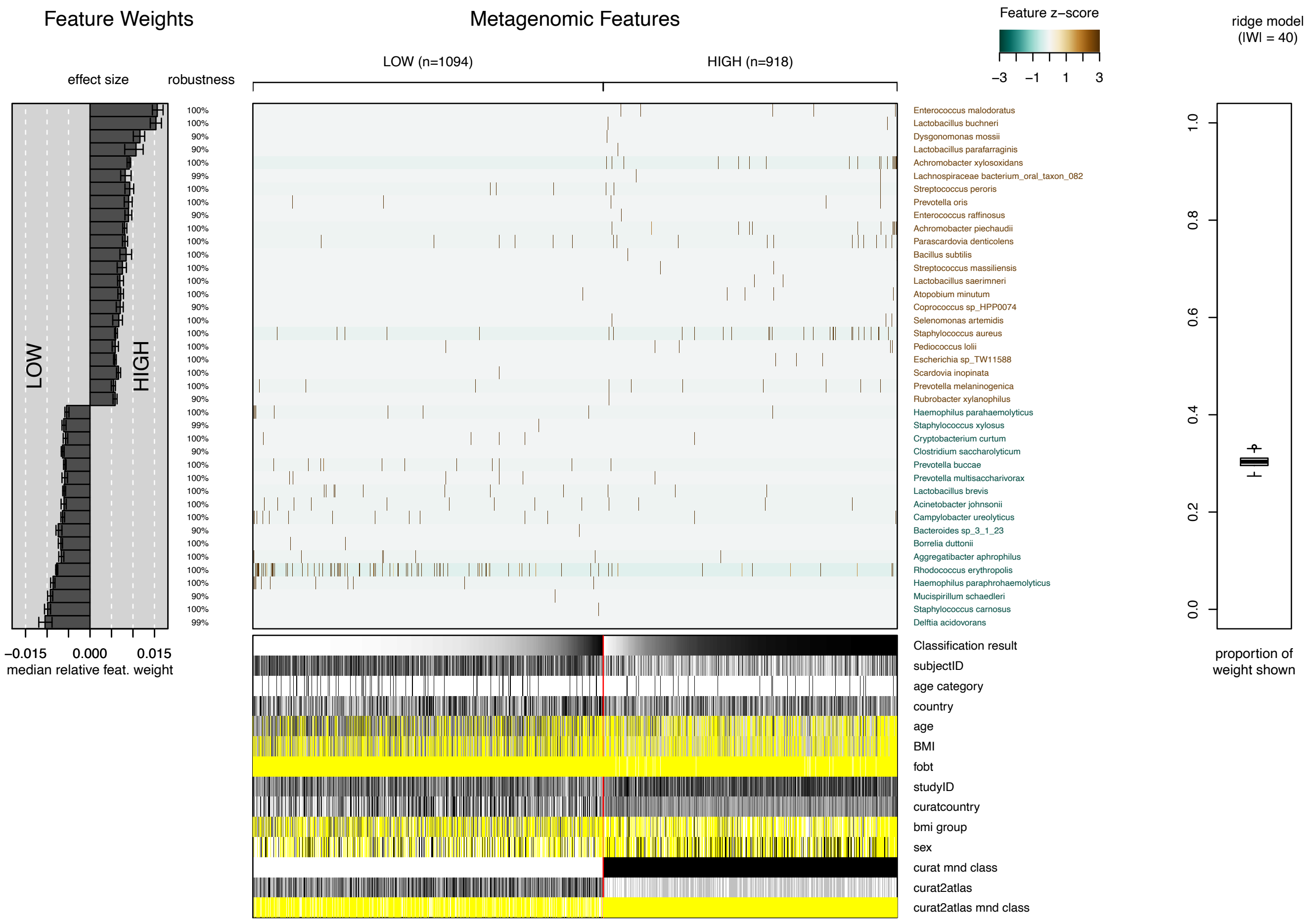

Supplementary Figure 4

Confounder Analysis of Variables Sex, BMI grouping and Age for Atlas Dataset (A) and Curated Dataset (B). Note the Label here is AD disease grouping. The conditional entropy check serves to identify any interdependence of confounders on each other. It quantifies the unique information contained in one variable with respect to another, with a value of 0 highlighting identical nonsensical variables, none of which are identified here for both datasets. Single covariate logistic regression analysis and Fisher tests were used to identify any correlation between the metadata variables age, sex and BMI grouping with the label AD disease burden. These analyses show that the male sex was correlated with increased AD burden in both datasets with a significant Fisher test p value in both datasets ($p<0.001$). However, age and BMI had opposite findings in the two datasets, both with significant correlation (Fisher test $p<0.001$). All three metadata variables were further investigated to check if they had confounding effects on individual microbial features. The SIAMCAT package does this by visualising the variance explained by the label (in this case AD disease grouping) compared to the variance explained by each metadata variable. Variables with many features in the upper left corner suggest that they confound the label associations but were not identified here in either dataset.

Supplementary Figure 4

A

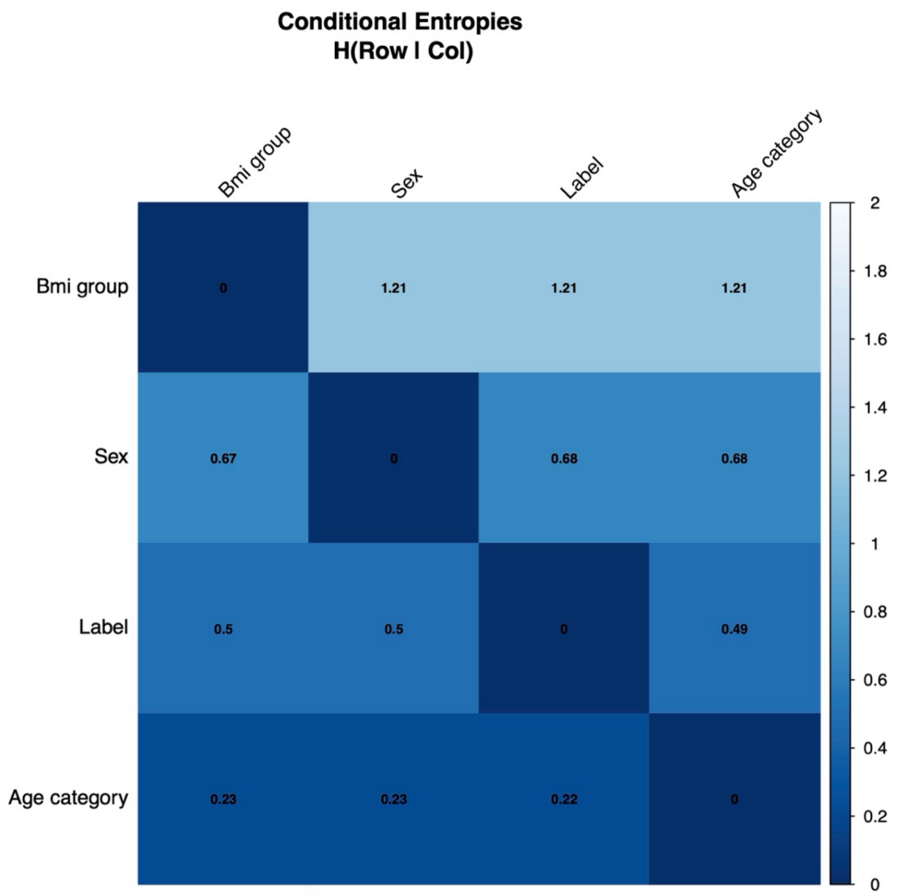

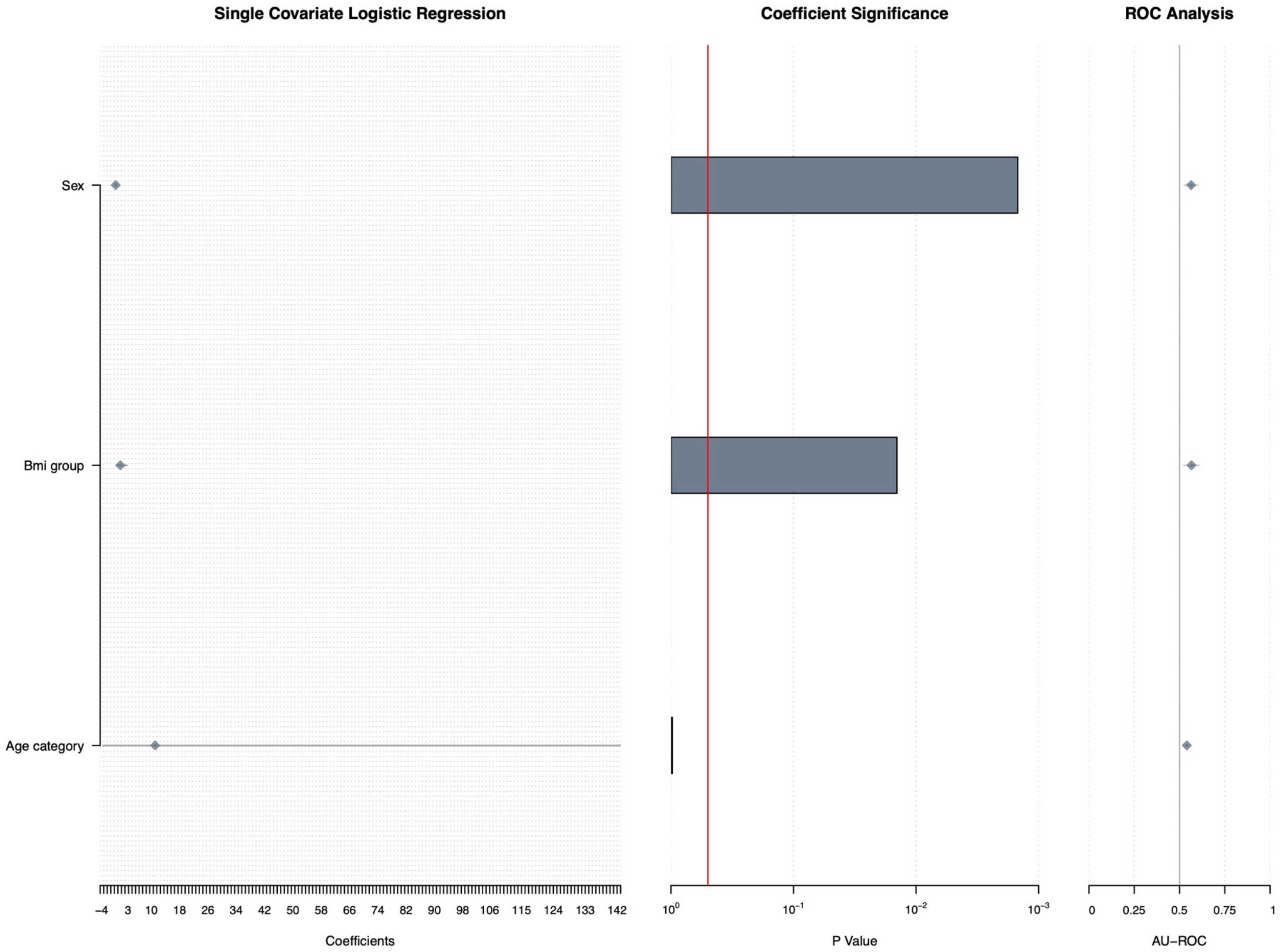
Single Covariate Logistic Regression
Coefficient Significance
ROC Analysis
Sex
Bmi group
Age category
−4 3 10 18 26 34 42 50 58 66 74 82 90 98 106 115 124 133 142
Coefficients
10^{0} 10^{-1} 10^{-2} 10^{-3}
P Value
0 0.25 0.5 0.75 1
AU−ROC

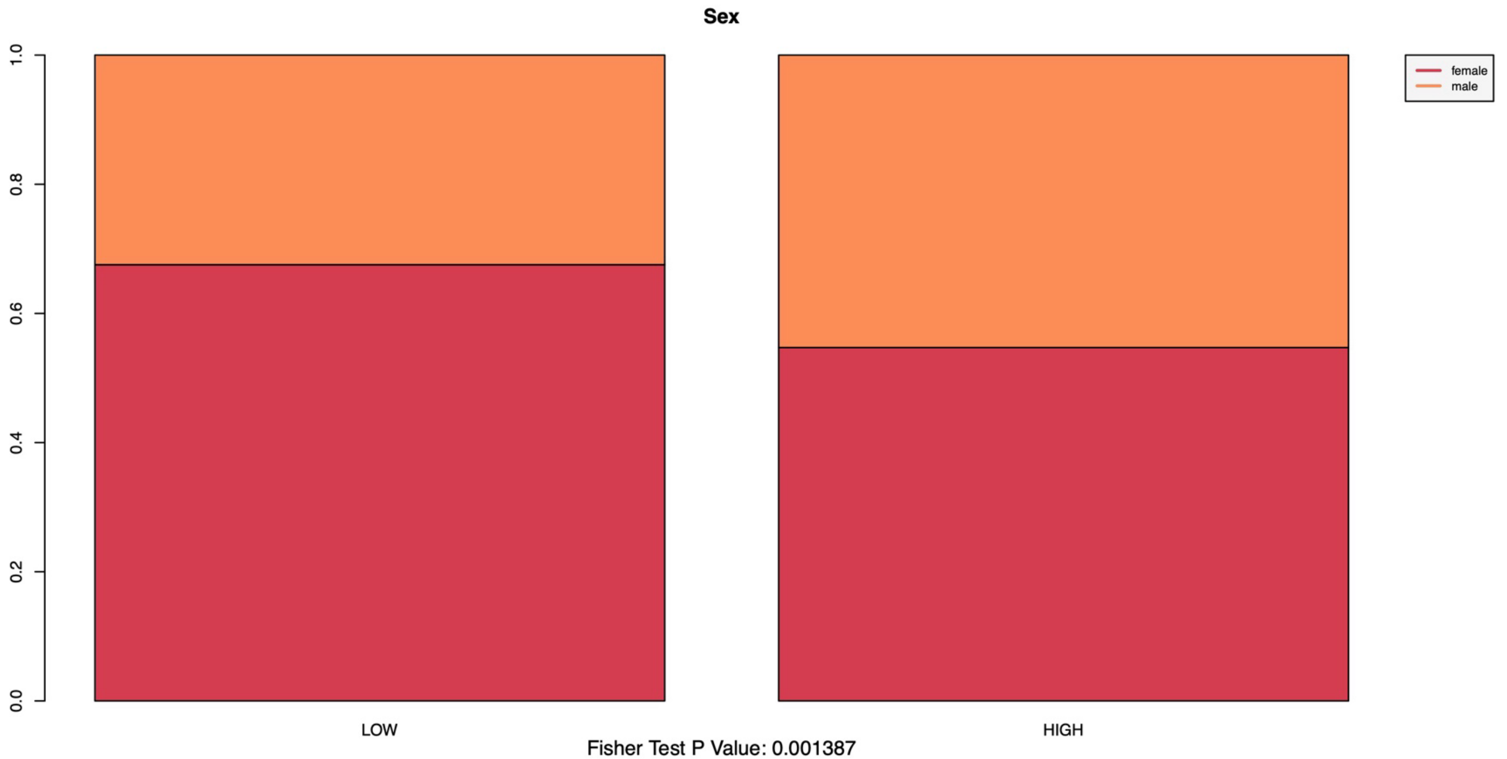

Fisher Test P Value: 0.001387

	LOW	HIGH	Sum
female	129	412	541
male	62	341	403
Sum	191	753	944

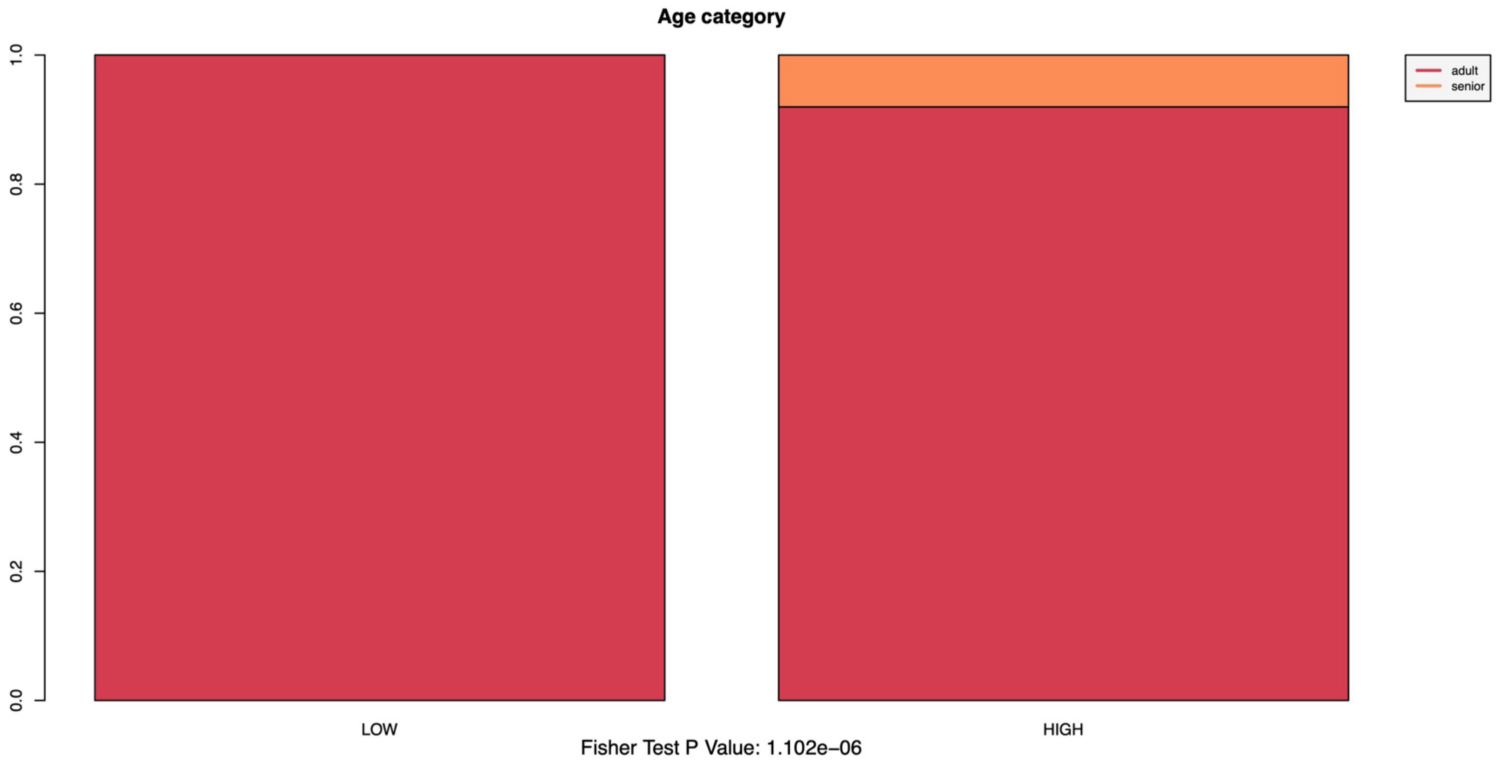

	LOW	HIGH	Sum
adult	191	675	866
senior	0	59	59
Sum	191	734	925

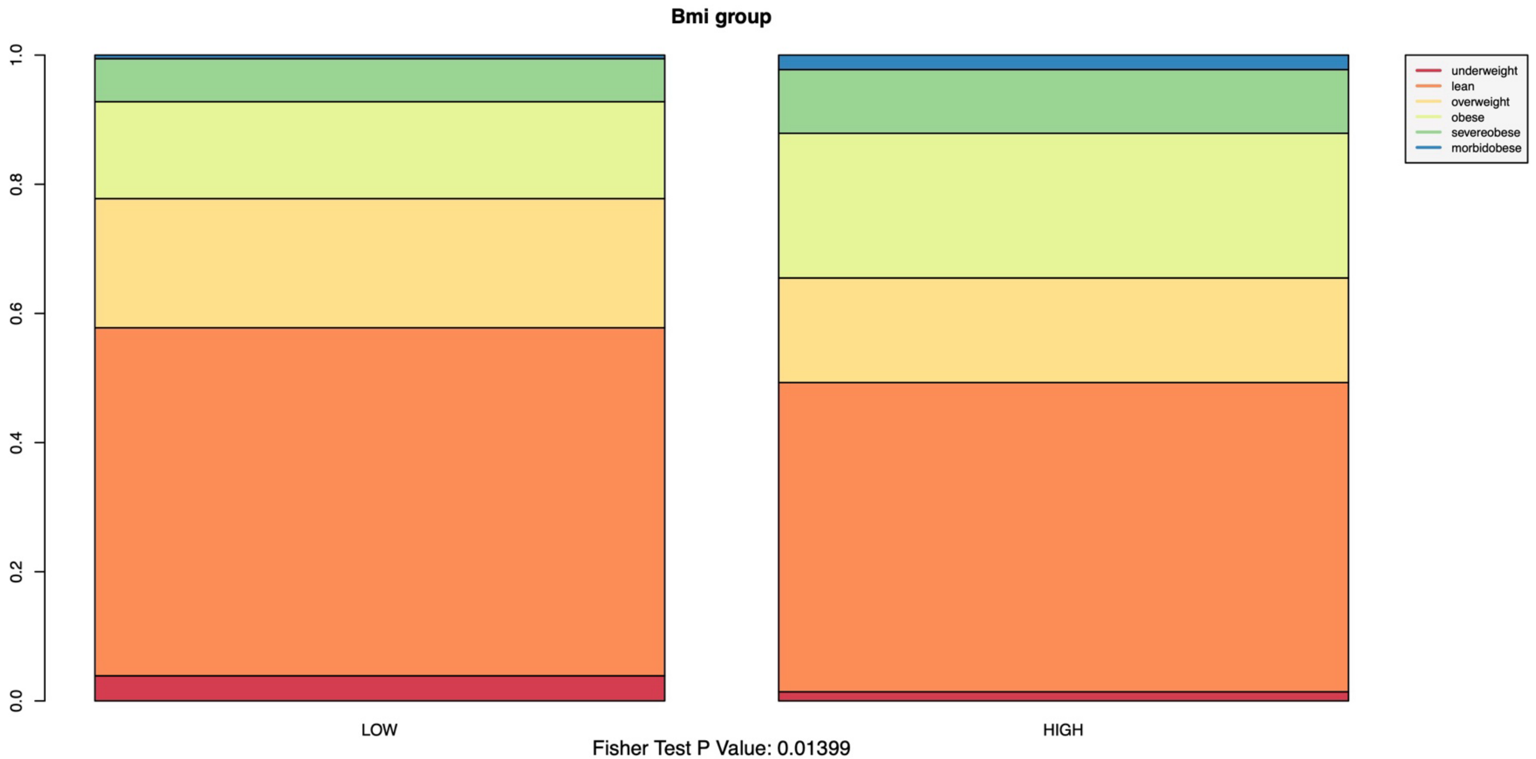

Fisher Test P Value: 0.01399

	LOW	HIGH	Sum
underweight	7	10	17
lean	97	340	437
overweight	36	115	151
obese	27	159	186
severeobese	12	70	82
morbidobese	1	16	17
Sum	180	710	890

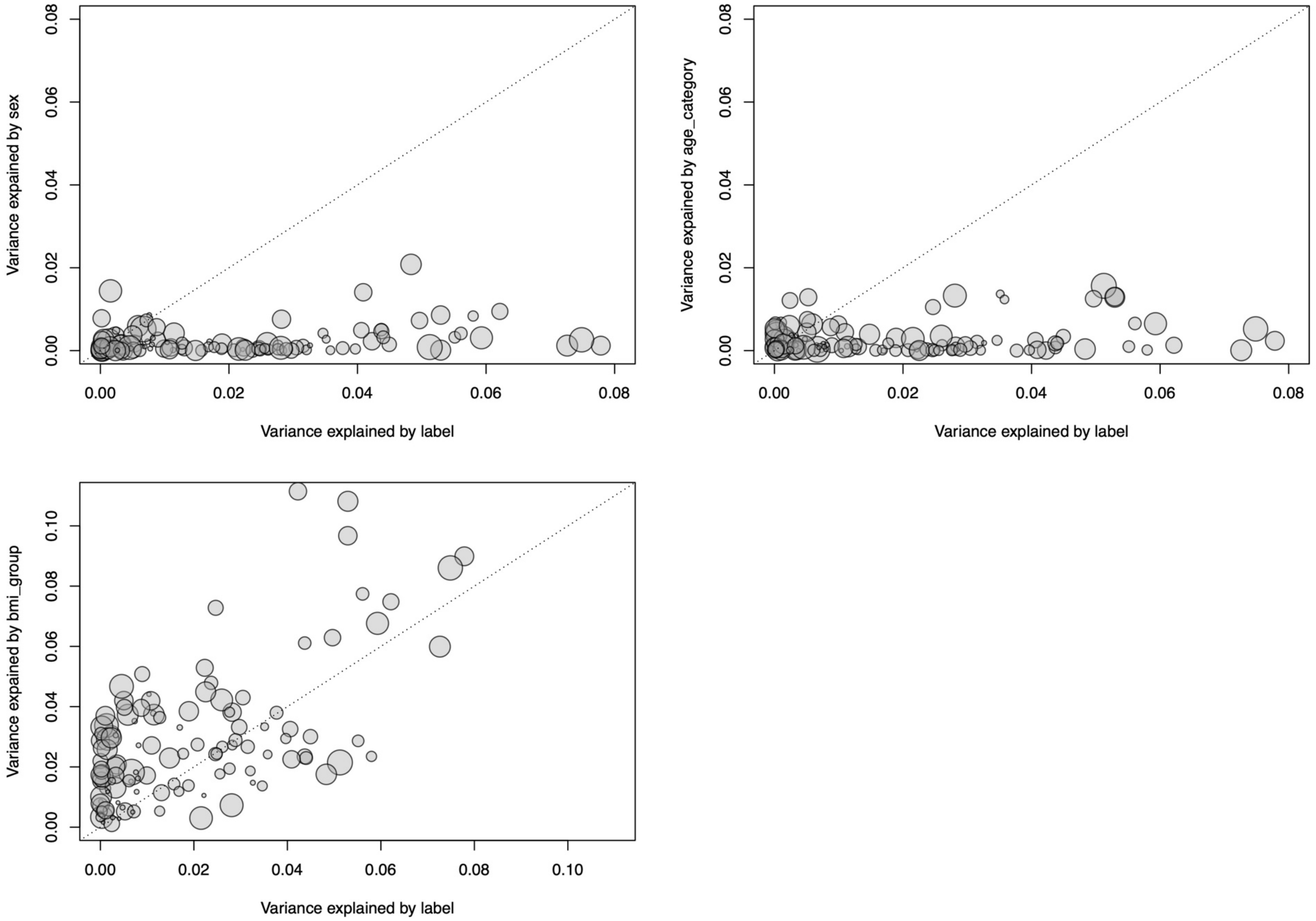
Variance expained by sex
Variance explained by label
Variance expained by age_category
Variance explained by label
Variance expained by bmi_group
Variance explained by label

Supplementary Figure 4

B

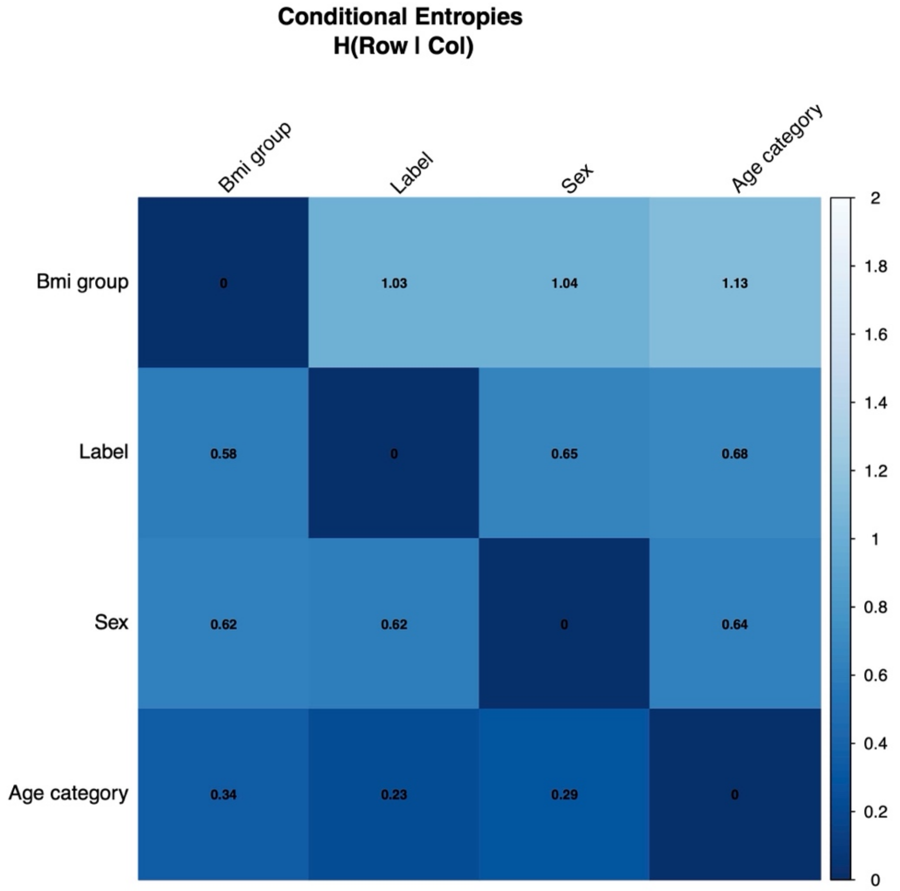

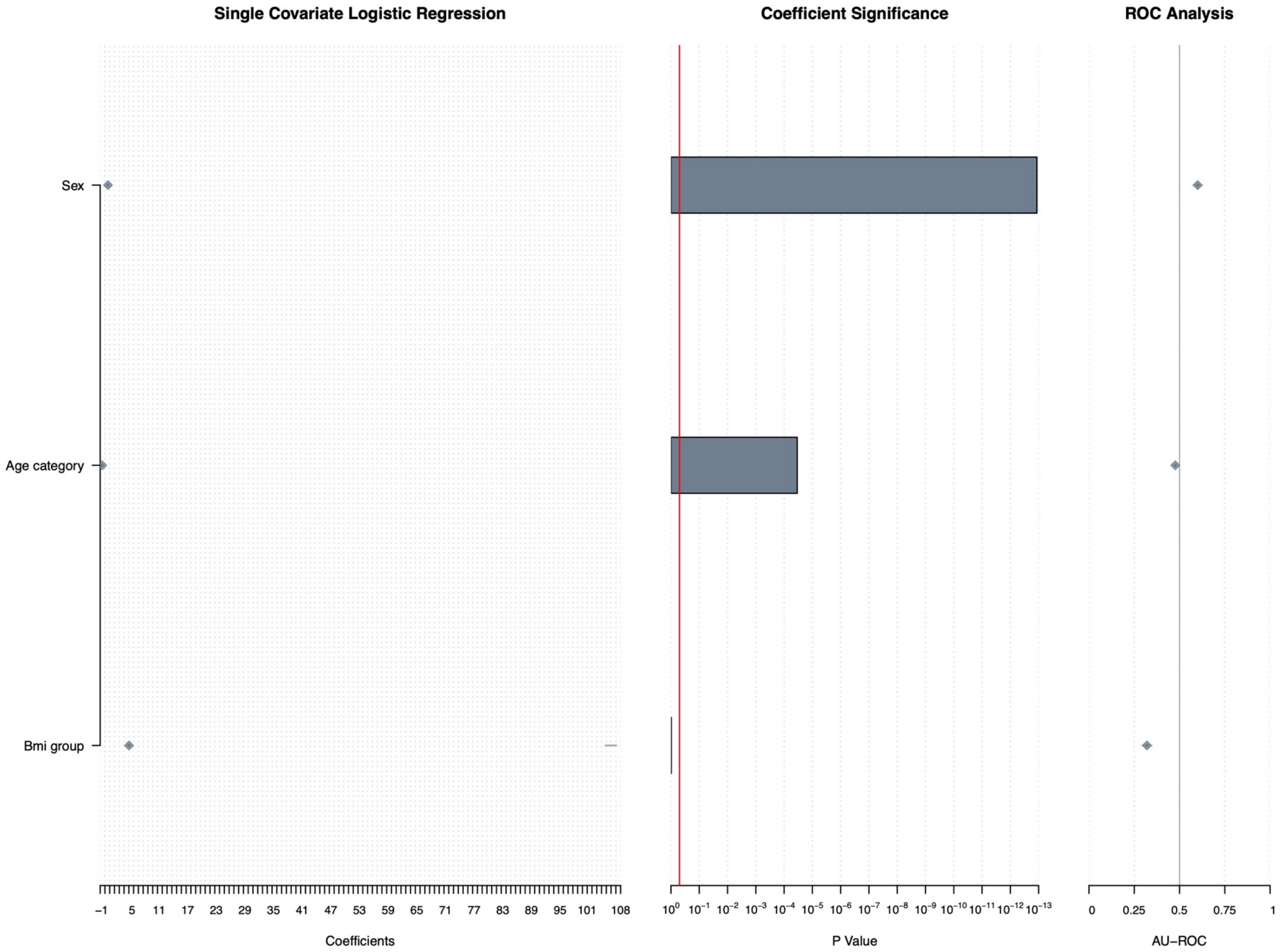

Single Covariate Logistic Regression
Coefficient Significance
ROC Analysis
Sex
Age category
Bmi group
−1 5 11 17 23 29 35 41 47 53 59 65 71 77 83 89 95 101 108
Coefficients
10^0 10^{-1} 10^{-2} 10^{-3} 10^{-4} 10^{-5} 10^{-6} 10^{-7} 10^{-8} 10^{-9} 10^{-10} 10^{-11} 10^{-12} 10^{-13}
P Value
0 0.25 0.5 0.75 1
AU−ROC

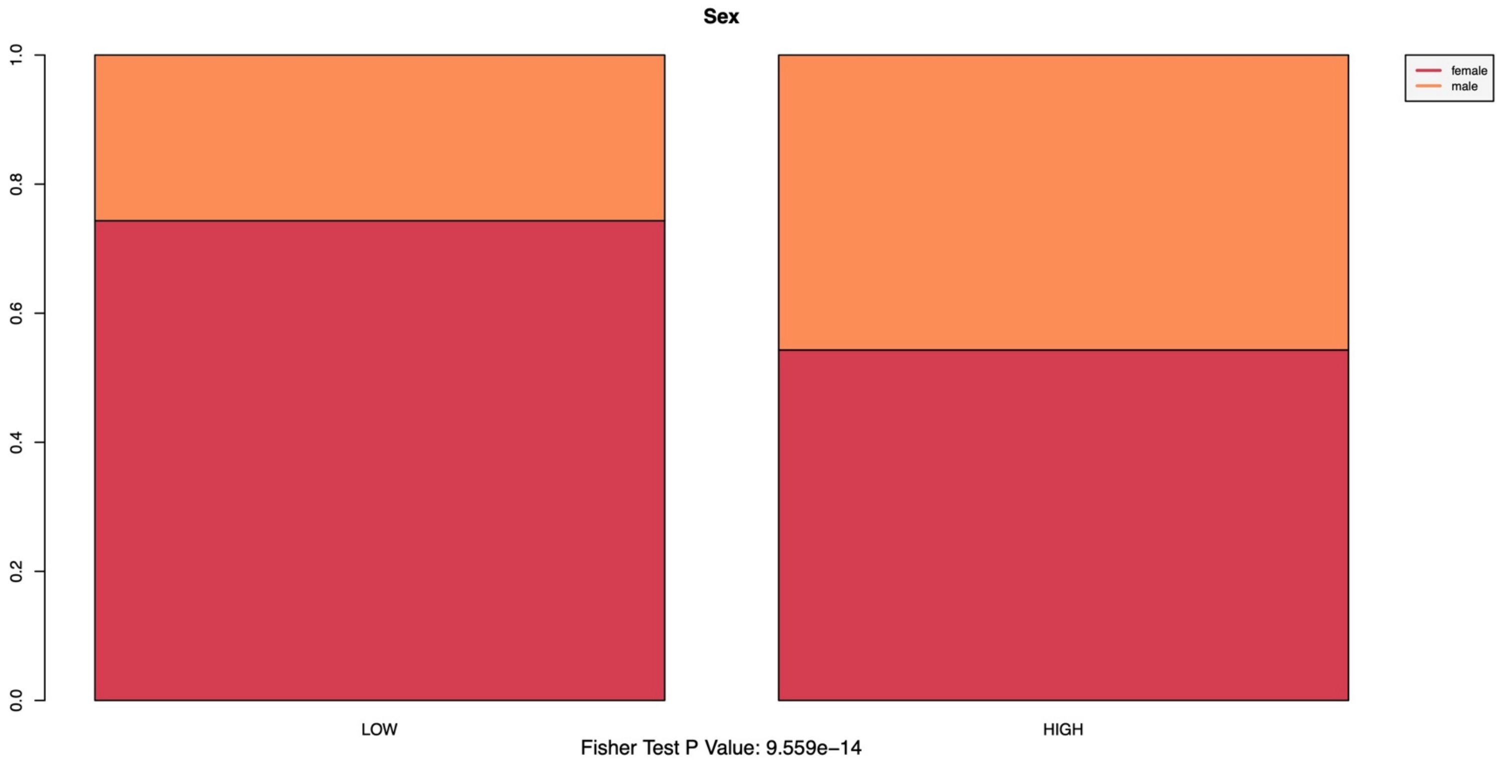

	LOW	HIGH	Sum
female	582	284	866
male	201	239	440
Sum	783	523	1306

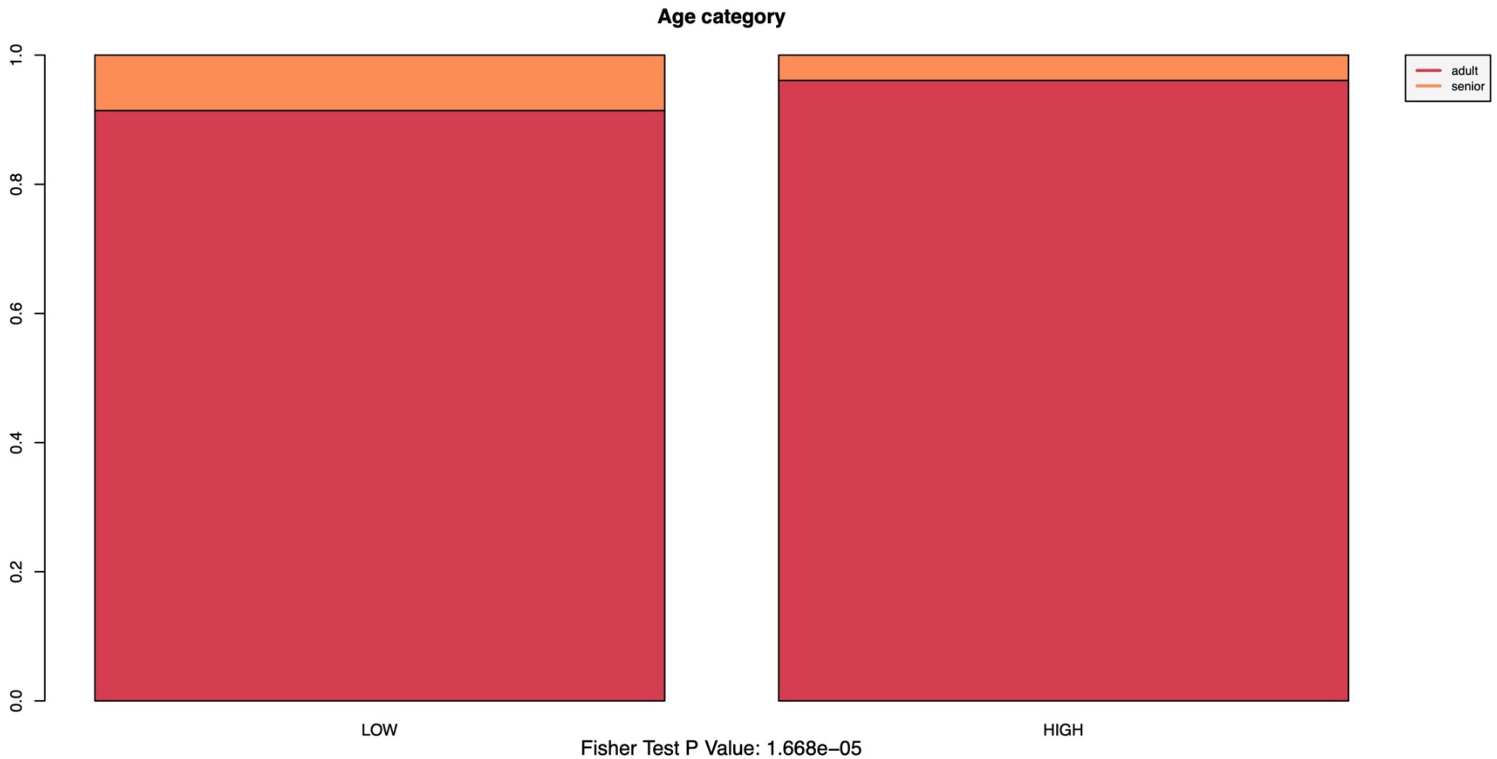

Fisher Test P Value: 1.668e−05

	**LOW**	**HIGH**	**Sum**
adult	1000	882	1882
senior	94	36	130
Sum	1094	918	2012

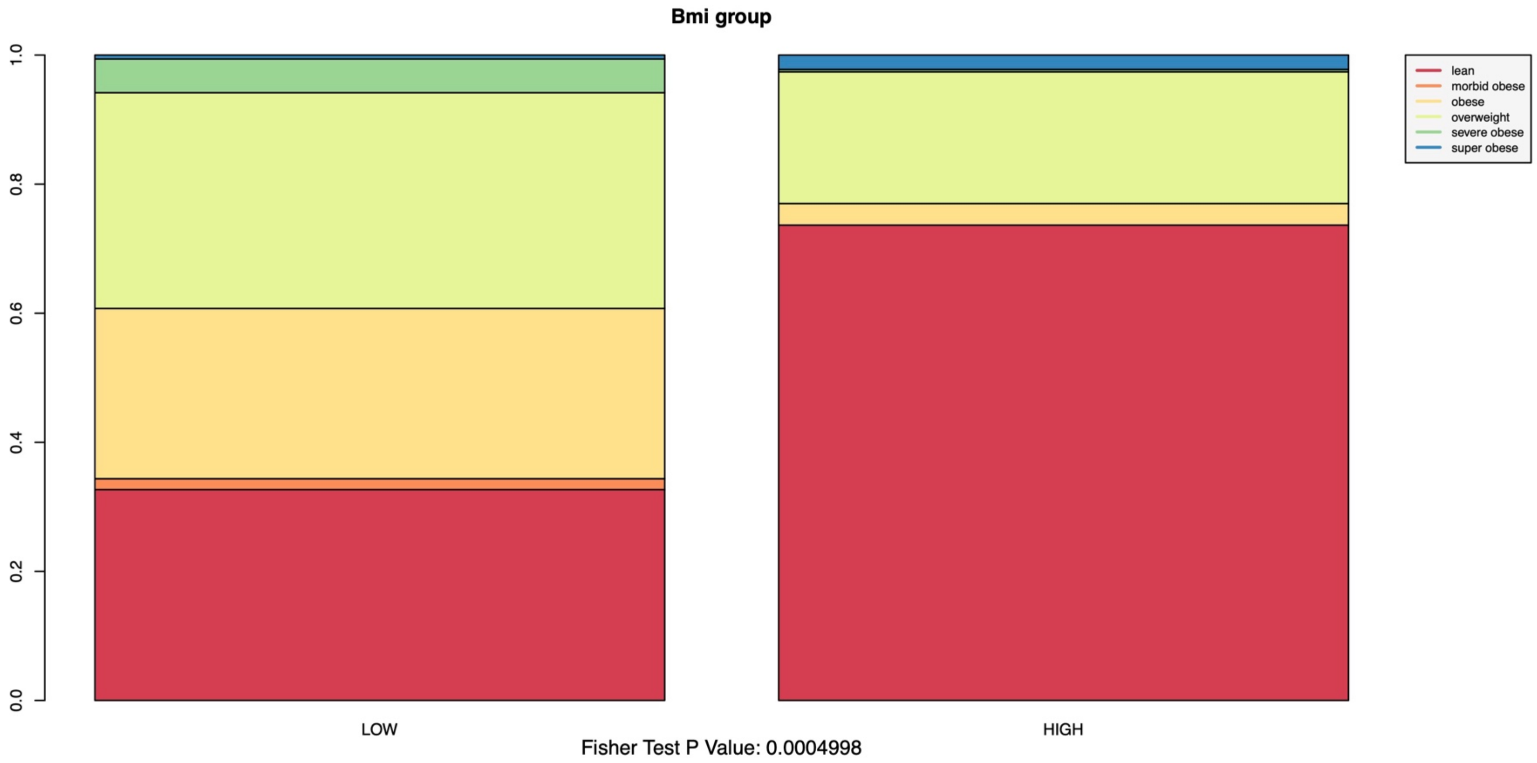

	**LOW**	**HIGH**	**Sum**
lean	213	397	610
morbid obese	11	0	11
obese	172	18	190
overweight	218	110	328
severe obese	34	2	36
super obese	4	12	16
Sum	652	539	1191

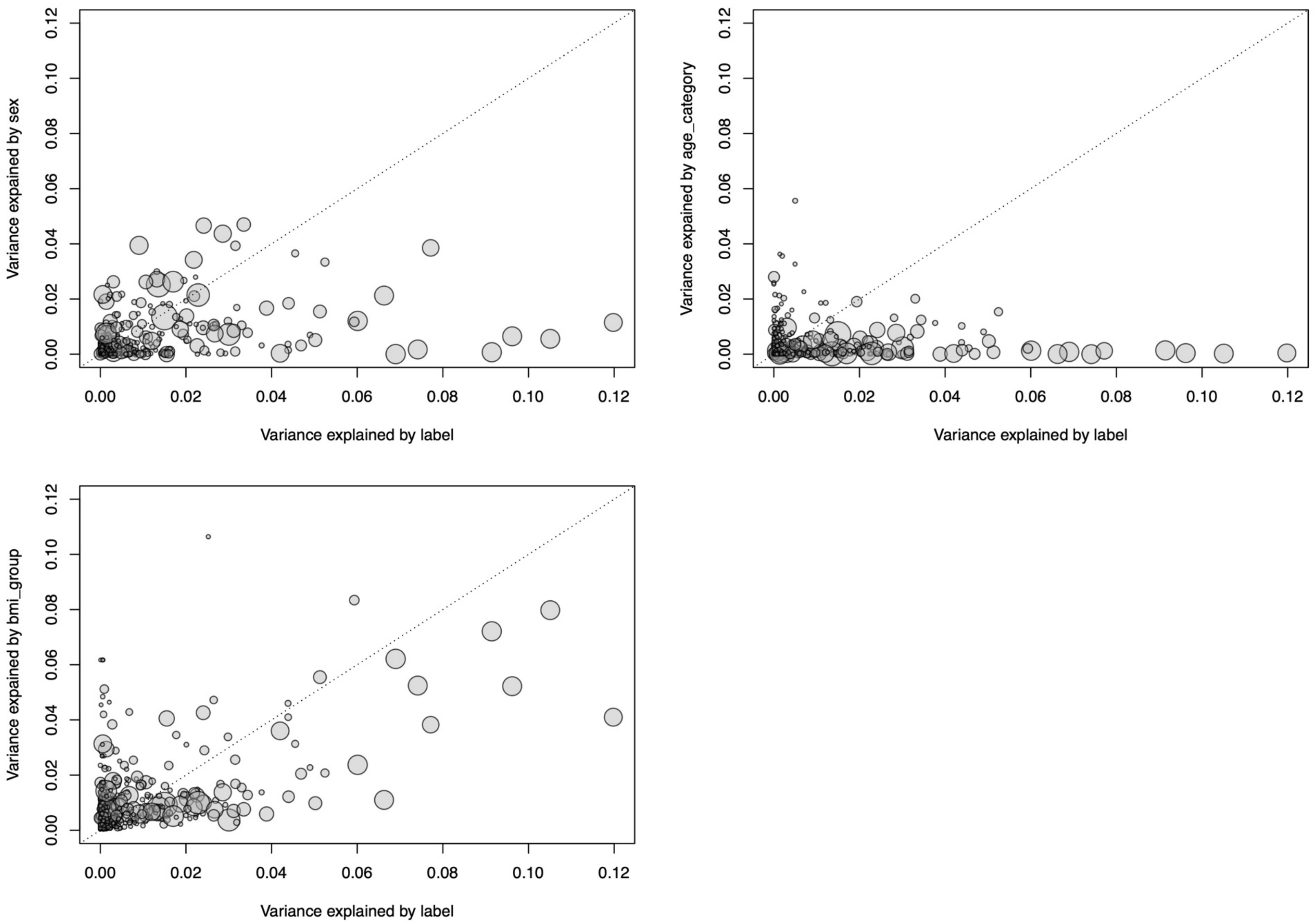
Variance expained by sex
Variance explained by label
Variance expained by age_category
Variance explained by label
Variance expained by bmi_group
Variance explained by label
